# Comparative transcriptome and coexpression network analysis of carpel quantitative variation in *Paeonia rockii*

**DOI:** 10.1186/s12864-019-6036-z

**Published:** 2019-08-29

**Authors:** Na Liu, Fangyun Cheng, Yuan Zhong, Xin Guo

**Affiliations:** Peony International Institute, Beijing Key Laboratory of Ornamental Plants Germplasm Innovation & Molecular Breeding, National Engineering Research Center for Floriculture, Beijing Laboratory of Urban and Rural Ecological Environment, Key Laboratory of Genetics and Breeding in Forest Trees and Ornamental Plants of Ministry of Education, School of Landscape Architecture, Beijing Forestry University, Beijing, 100083 China

**Keywords:** *Paeonia rockii*, Carpel quantitative variation, Comparative transcriptome, Clustering pattern, Weighted gene coexpression network

## Abstract

**Background:**

Quantitative variation of floral organs in plants is caused by an extremely complex process of transcriptional regulation. Despite progress in model plants, the molecular mechanisms of quantitative variation remain unknown in woody flower plants. The *Paeonia rockii* originated in China is a precious woody plant with ornamental, medicinal and oil properties. There is a wide variation in the number of carpel in *P. rockii*, but the molecular mechanism of the variation has rarely been studied. Then a comparative transcriptome was performed among two cultivars of *P. rockii* with different development patterns of carpel in this study.

**Results:**

Through the next-generation and single-molecule long-read sequencing (NGS and SMLRS), 66,563 unigenes and 28,155 differentially expressed genes (DEGs) were identified in *P. rockii*. Then clustering pattern and weighted gene coexpression network analysis (WGCNA) indicated that 15 candidate genes were likely involved in the carpel quantitative variation, including floral organ development, transcriptional regulatory and enzyme-like factors. Moreover, transcription factors (TFs) from the MYB, WD, RING1 and LRR gene families suggested the important roles in the management of the upstream genes. Among them, *PsMYB114-like*, *PsMYB12* and *PsMYB61-like* from the MYB gene family were probably the main characters that regulated the carpel quantitative variation. Further, a hypothetical model for the regulation pattern of carpel quantitative variation was proposed in which the candidate genes function synergistically the quantitative variation process.

**Conclusions:**

We present the high-quality sequencing products in *P. rockii*. Our results summarize a valuable collective of gene expression profiles characterizing the carpel quantitative variation. The DEGs are candidate for functional analyses of genes regulating the carpel quantitative variation in tree peonies, which provide a precious resource that reveals the molecular mechanism of carpel quantitative variation in other woody flower crops.

**Electronic supplementary material:**

The online version of this article (10.1186/s12864-019-6036-z) contains supplementary material, which is available to authorized users.

## Background

As an indispensable part of the human diet, edible oil provides us with an abundant amount of fat and energy. At present, woody oil crops have become an important source of human edible oil because of their high nutritional value, strong resistance and stable yield [1, 2]. For woody oil crops, yield is the direct embodiment of production value. And the development of floral organs directly affects the yield and reproduction of seeds, of which the number of carpels or fruits is certainly significant to the formation of yield. Tree peony, which belongs to the *Paeonia* section *Moutan* DC., Paeoniaceae, is a peculiar resource for ornamental and medicinal cultivation in China. In recent years, it is also considered as a valuable emerging woody oil crop with high unsaturated fatty acid (approximately 90%) and α-linolenic acid (approximately 40%) contents in seeds [3]. *P. rockii*, one of the tree peony species, is endemic to the Qinling Mountains and adjacent areas in central China. It has been cultivated for more than 1600 years and has the greatest numbers of extant plants of all tree peony species. It is also one of the most important ancestral species of cultivated tree peonies. The wild species of *P. rockii* are mainly distributed in Gansu, Shanxi, Henan and Hubei provinces of China. After a long period of introduction and domestication, hybridization and selective breeding, the origin and evolution center of cultivated varieties (groups) has been formed mainly in Gansu province, and distributed to other areas in northwest China such as Qinghai and Shanxi provinces. At present, because of its strong resistences to drought and cold, *P. rockii* has shown great potential for mass cultivation as a valuable emerging woody oil crop in China, as well as being cultivated in many other countries in Asia, America, Europe and Oceania [4, 5]. In the process of its expansion and development, how to increase the seed yield has become a key issue. The fruit of tree peony including *P. rockii* is aggregate follicle composed of various follicles, in which every follicle is developed from a single carpel and carpel quantitative variation is ubiquitous. Because the carpel number affects seed yield directly, comprehensive analysis of the molecular mechanisms driving carpel quantitative variation is of great significance for high yield breeding.

The continuous improvement of ABCDE flower organ development model and the four-factor model provides an important basis for exploring the molecular mechanism of carpel quantitative variation in plants including *P. rockii*. Carpel development is closely related to the CDE-like functional genes [6–14]. In *Arabidopsis thaliana*, single or simultaneous mutations of the D-class genes *STK*, *SHP1* and *SHP2* can transform part of the ovule into carpeloid structures [15, 16]; the co-overexpressed AG-SEP3 can transform nutrient leaves into carpels [17]. *MOSAIC FLORAL ORGANS1* (*MFO1*/ *MADS6*) is an *AGL6*-like gene in *Oryza sativa*. In the flower of *mfo1* mutants, the determinacy of the floral meristem was lost and extra carpels or spikelets developed in the *mfo1* florets [18]. In *Zea mays*, the gene *bde* is a member of the *AGL6* family of MIKC-type TFs, which is sister to the SEP clade [19, 20]. Mutants of *bde* and *zag1* both produce extra carpels in female florets, besides BDE and ZAG1 interact in a complex that regulates the floral organ number [21]. The expression levels of *VvAG1*, *VvAP1*, *VvAP2*, *VvCLV1*, *VvCLV2*, *VvSEP3* and *VvSPT* were higher in tricarpellate ovaries than in bicarpellate ovaries in ‘Xiangfei’ grapevines (*Vitis vinifera*) [22]. In addition, studies have also identified other determinants of carpel quantitative variation. In *A. thaliana*, *AtCLAVATA1*, *AtCLAVATA2* and *AtCLAVATA3* mainly regulate the size of floral meristems and the number of floral organs [23–27]; AtRING1 regulates stem cell-determining carpel development mainly through repression of class I *KNOX* genes, and indeterminate carpel growth in the *atring1a*; *atring1b* mutant is associated with homeotic replumto-carpel and ovule-to-carpel conversions [28]; *ant ail7* double mutants produce increased numbers of carpels, which have defects in valve fusion and a loss of apical tissues [29]. Studies in *Cucumis sativus* have shown that *CLAVATA3* is the optimal candidate gene for regulating the carpel number [30]. In *Brassica rapa*, it was proven that the multilocular mutant contained more stamens and carpels in the functional characterization of the multilocular silique gene *BrCLV3*, and most of its siliques had 4 locules with a shorter, rounder and thicker shape and extra gynoecium inside [31]. In *P. rockii*, the SSR marker loci associated with carpel number, such as PS242, PS180 and PS290, have been studied in our laboratory, but an in-depth and comprehensive study on the carpel quantitative variation has not been reported.

RNA-seq technology is an important method to obtain effective functional genes in crops, especially for crops without reference genomic information. At present, RNA-seq has been widely used to study floral organ development in woody plants [32–36]. With the development of high-throughput sequencing technology, numerous studies have been carried out by SMLRS or in combination with NGS technology. A study of the biosynthesis of tanshinone diterpenoids in *Salvia miltiorrhiza* demonstrates that tanshinone pigments are produced and accumulated in the root periderm applying a combination of NGS and SMRT sequencing to various root tissues, particularly including the periderm [37]. In *Sorghum bicolor*, the study reveals transcriptome-wide full-length isoforms at an unprecedented scale with over 11,000 novel splice isoforms by Pacific Biosciences single-molecule real-time long-read isoform sequencing. Additionally, APA of ~ 11,000 expressed genes and more than 2100 novel genes are uncovered [38]. When single-molecule sequencing technology was used in *Z. mays*, it produced 111,151 transcripts from 6 tissues capturing ~ 70% of the genes annotated in maize RefGen_v3 genome. It also identified a large number of novel long non-coding RNAs and fusion transcripts [39]. At present, only NGS and SLAF-seq technologies have been applied frequently in tree peonies [40–43]. It is the first attempt to explore the carpel quantitative variation by SMLRS.

WGCNA is a method for analyzing the expression patterns of multiple sample genes [44]. It adopts a weighted coexpression strategy (no scale distribution), which is more consistent with biological phenomena and show the interaction between genes. Additionally, the level of connectivity can reflect how well a gene is connected to other genes [45, 46]. At present, WGCNA has been combined with transcriptome for various plants. Genome-wide network model capturing seed germination reveals coordinated regulation of plant cellular phase transitions in *A. thaliana* [47]. Global transcriptome and coexpression network analyses are combined to reveal cultivar-specific molecular signatures associated with seed development and seed size/weight determination in chickpea [48]. Temporal network analysis identifies early physiological and transcriptomic indicators of mild drought in *B. rapa* [49]. This study will be the first attempt to explore the coexpression network relationship between the genes related to carpel quantitative variation in tree peonies.

At present, there are still no reports on the molecular mechanism of carpel quantitative variation in *Paeonia* including *P. rockii*. Here, we combined NGS and SMLRS technologies to detect two cultivars of *P. rockii*. Then we screened clustering patterns and modules of DEGs expressed specifically at critical stages to reveal transcriptome dynamics and transcriptional regulatory networks. Heatmaps and phylogenetic analysis of DEGs were performed to identify candidate genes involved in the carpel quantitative variation. This study provides a theoretical basis for understanding the regulation mechanism of carpel quantitative variation in tree peony and will be of great significance for the genetic improvement of yield traits of woody oil crops.

## Results

### Morphological description of flower bud differentiation in *P. rockii*

**Fig. 1:**
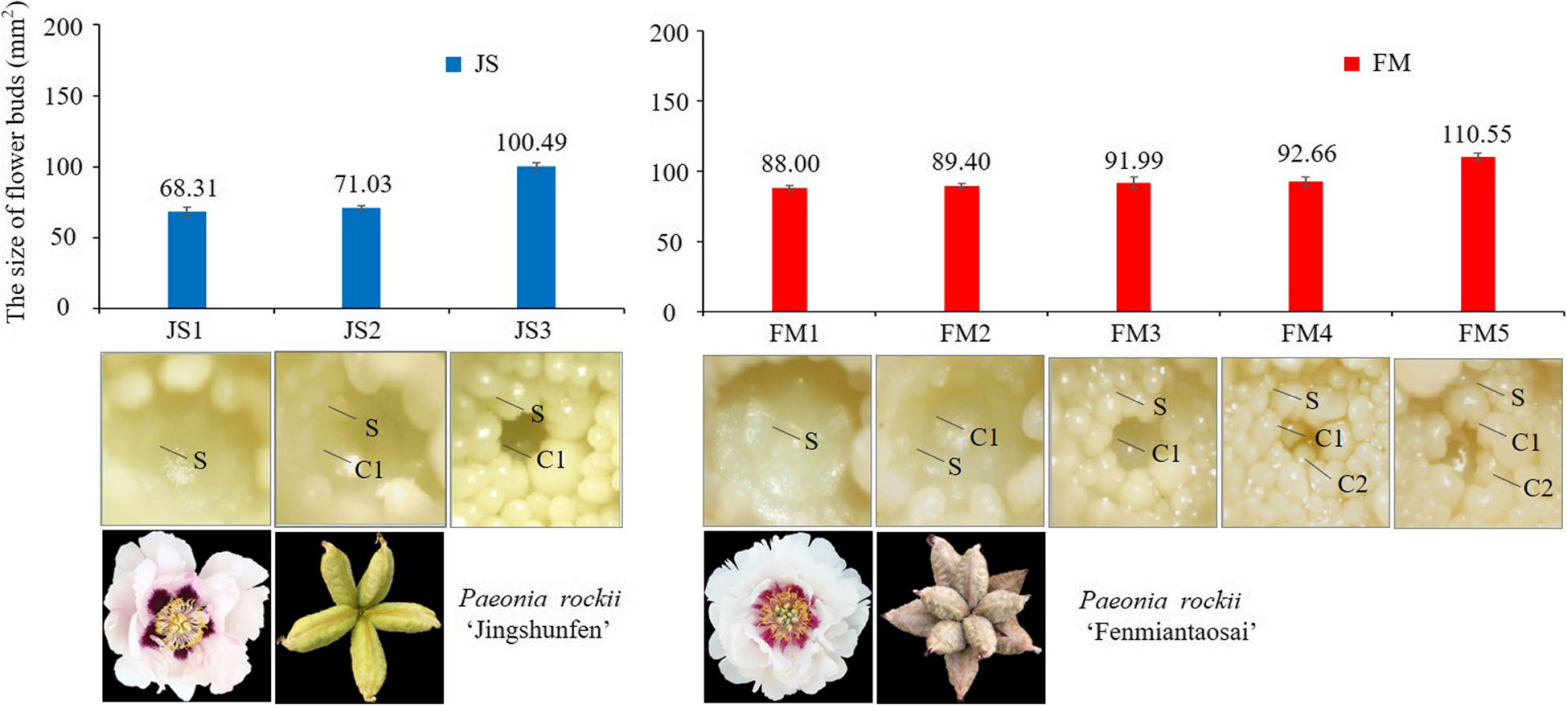
The size of flower buds and material collection stage for JS and FM. The size of each flower bud is expressed as the product of its length and diameter. **S** stamen primordium; **C1** The first whorl of carpel primordium; **C2** The second whorl of carpel primordium; **JS1, FM1** The stamen primordium stage; **JS2, FM2** The first whorl of carpel primordia initial appearance stage; **JS3, FM3** The first whorl of carpel primordia ventral suture formation stage; **FM4** The second whorl of carpel primordia initial appearance stage; **FM5** The second whorl of carpel primordia ventral suture formation stage

The process of differentiation and development of stamen and multiwhorl carpel primordia in tree peony has been studied in our laboratory. In this study, we screened two cultivars with significant difference in carpel number, *P. rockii* ‘Fenmiantaosai’ (FM) and *P. rockii* ‘Jingshunfen’ (JS), as shown in Fig. 1. The differences in the differentiation and development of carpel primordia between FM and JS were clear under an anatomical lens. In JS, there was one whorl of carpel primordia that could develop into 5 carpels, but there was a second whorl of carpel primordia generated inside the first whorl that had formed the ventral suture in FM. The carpel quantitative variation was presented in both whorls of FM. Generally, there were 5–8 carpels in the first whorl and 1–5 in the second whorl, so FM generated more carpels than JS. We also observed that the size of flower buds both in JS and FM increased in the process of flower bud differentiation, but the average size of flower buds at 1–3 stages in FM was always larger than that in JS (Fig. 1).

### Datasets evaluation

**Fig. 2:**
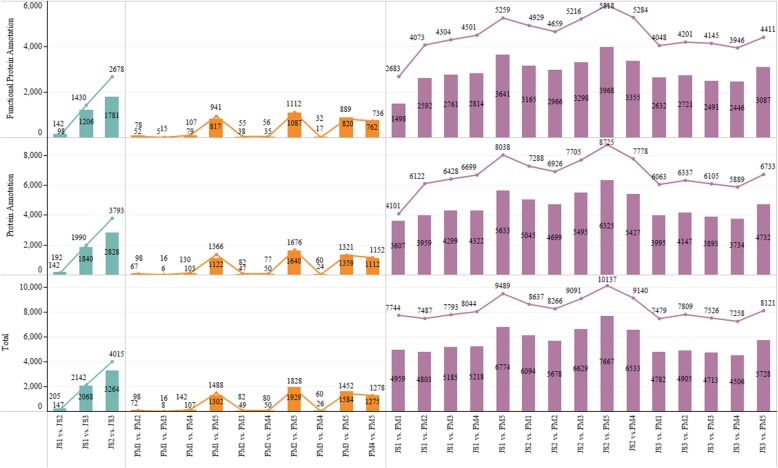
The number and protein annotation of DEGs in each comparison. Line graphs correspond to the upregulated DEGs. Bar graphs correspond to the downregulated DEGs. The numbers in the column represent the quantity of functional protein annotation, protein annotation and total DEGs. Comparisons in the row contrast JS1–3, FM1–5 and JS1–3 vs. FM1–5

A total of 1,200,796,738 clean reads and 169,822,068,623 clean bases were obtained in the NGS method. The SMLRS method produced a total of 22.00 G data, and the average length of each cell was 19.80 K, 19.00 K, 22.00 K, 16.30 K and 20.80 K, respectively. The average read length of insertion of the 1~2 K library was 1418 bp, 2~3 K library was 2415 bp, and > 3 K library was 3452 bp (Additional file 1). After correction by Quiver, 59,691 sequences with an accuracy greater than 0.99 and 215,592 sequences with an accuracy less than 0.99 were obtained. Then through the CD-HIT cluster analysis, 179,134 isoforms and 66,563 unigenes were obtained. Totally, approximately 60% of unigenes were 1000~3500 bp in lengh and 53,817 (80.85%) unigenes were functionally annotated (Additional file 2). Moreover, we found that when 3.75 < RPKM< 15 and RPKM> 15, there were more unigenes in FM than in JS (Additional file 3). The correlation between two accessions of the same cultivar was greater than that of different cultivars (Additional file 4). Further, a total of 28,155 DEGs were identified, and the number of DEGs in the two cultivars increased first and then decreased in the progress of flower bud differentiation. The number of DEGs between two accessions of the same cultivar was significantly less than that of different cultivars, which was consistent with the correlation test between accessions (Fig. 2). This indicated that the sequencing results were accurate, and the unigenes could be used for subsequent biological analysis.

### Clustering patterns of DEGs

**Fig. 3:**
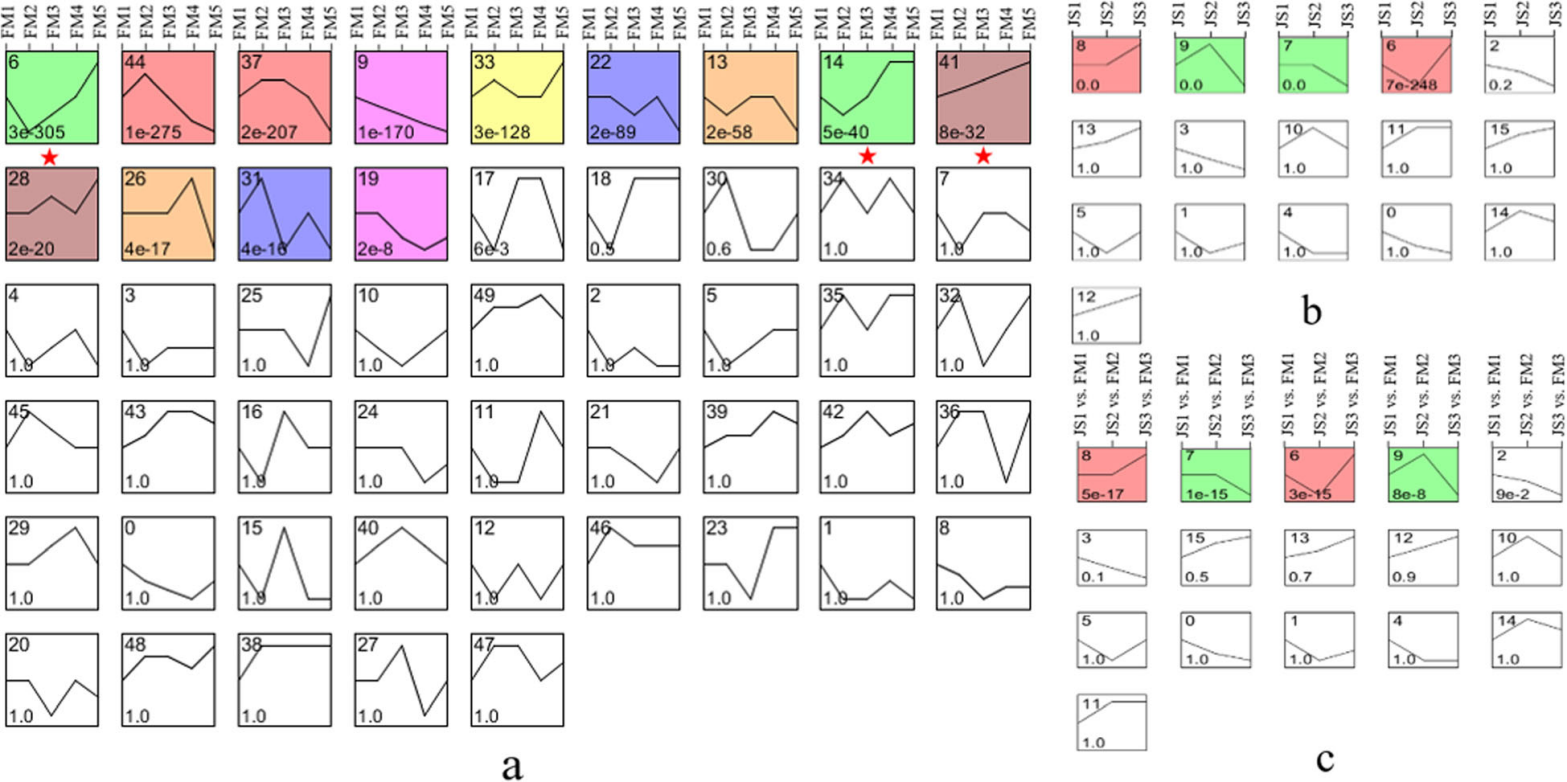
Clustering patterns of DEGs. **a** Clustering of FM gene expression profiles. The significant DEG enrichment patterns (profile 6, profile 14 and profile 41) are marked with asterisks. **b**, **c** Clustering of JS and JS vs. FM gene expression profiles. Clusters are ordered based on the number of genes and the profiles are ordered by significance. X axis represents the stages of FM1–5 **a**, JS1–3 **b** and JS1–3 vs. FM1–3 **c**, respectively. Y axis corresponds to the expression change of DEGs. The fold lines represent the expression trends of DEGs. The number in the upper left corner represents the serial number of the specific expression pattern, and the number in the lower left corner represents the *P*-value of the clustering pattern. The remarkable profiles represent the significantly enriched expression patterns

The clustering pattern of all DEGs were divided into 16 types in JS, 50 types in FM and 16 types for JS vs. FM. Then we observed that profile 6, 14 and 41 in FM were significant enrichment patterns, in which the expression levels of DEGs at the key stage (FM3 vs. FM4) was consistently upregulated, these DEGs could be considered as important candidate genes associated with carpel quantitative variation. Combining the different clustering patterns of JS and FM, we found that some DEGs were expressed in both cultivars but showed different expression patterns at the key stages. As a result, different combinations of these clustering patterns were used to screen and identify candidate genes. Additionally, JS1–3 and FM 1–3 were the same stages in both cultivars, but FM 1–3 showed carpel quantitative variation. Then we observed that some DEGs expressed in both JS1–3 and FM1–3 were consistently upregulated or downregulated in JS1–3 vs. FM1–3, so those could be considered as important regulators (Fig. 3).

### Analysis of FM clustering patterns

Profile 41 in FM was particularly noticeable at all sampling stages, in which the expression levels of DEGs at FM1–5 stages were consistently upregulated (Fig. 3a). In profile 41, there were 395 DEGs. The enriched gene ontology (GO) items were mainly relative to catalytic activity, single tissue metabolism, oxidoreductase activity and redox process. The enriched kyoto encyclopedia of genes and genomes (KEGG) pathways mainly included carbon metabolism and mobilization pathways, amino acid synthesis, glycolysis and gluconeogenesis (Additional file 5). Then we identified 4 MADS-box genes (*PsSEP3/AGL9*, *PsAGL9*, *PsPI2* and *PsAP3*) and 7 TFs from 5 gene families (*PsNAC17*, *PsPsWRAP73*, *PsHVA22–1*, *PsHVA22–2*, *PsMYB12*, *PsTCP4* and *PsTCP2-like*) (Additional file 6).

Profile 14 in FM was particularly noticeable during the intermediate sampling stages, in which the expression levels of DEGs at FM3 and FM4 stages were consistently upregulated (Fig. 3a). In profile 14, there were 322 DEGs. The enriched GO items were relative to exopeptidase activity and carboxypeptidase activity, and the enriched KEGG pathway was proteasome (Additional file 7). Then we identified 5 MADS-box genes (*PsAGL104*, *PsAGL6–1*, *PsAGL12*, *PsAGL6–2* and *PsAP1*), 1 enzyme-like gene (*PsLRR receptor-like*) and 3 TFs from 3 gene families (*PsBHLH-2*, *PsB3* and *PsAIL1*) (Additional file 6).

Profile 6 in FM was particularly noticeable during the later sampling stages, in which the expression levels of the DEGs at FM3, FM4 and FM5 stages were significantly consistently upregulated (Fig. 3a). In profile 6, there were 1269 DEGs. The enriched GO items were mainly relative to regulation of cellular process, responses to chemicals, and active transmembrane transporter activity, and the pathways enriched in KEGG were mainly associated with starch and sucrose metabolism, circadian rhythm-plant, and ribosome biogenesis in eukaryotes (Additional file 8). Then 1 MADS-box gene (*PsAG*), 2 enzyme-like genes (*LRR receptor-like FEI1* and *RING1-like*) and 23 TFs from 8 gene families (*PsERF073*, *PsMYB56*, *PsGATA8-like* and so on) were identified (Additional file 6).

**Fig. 4:**
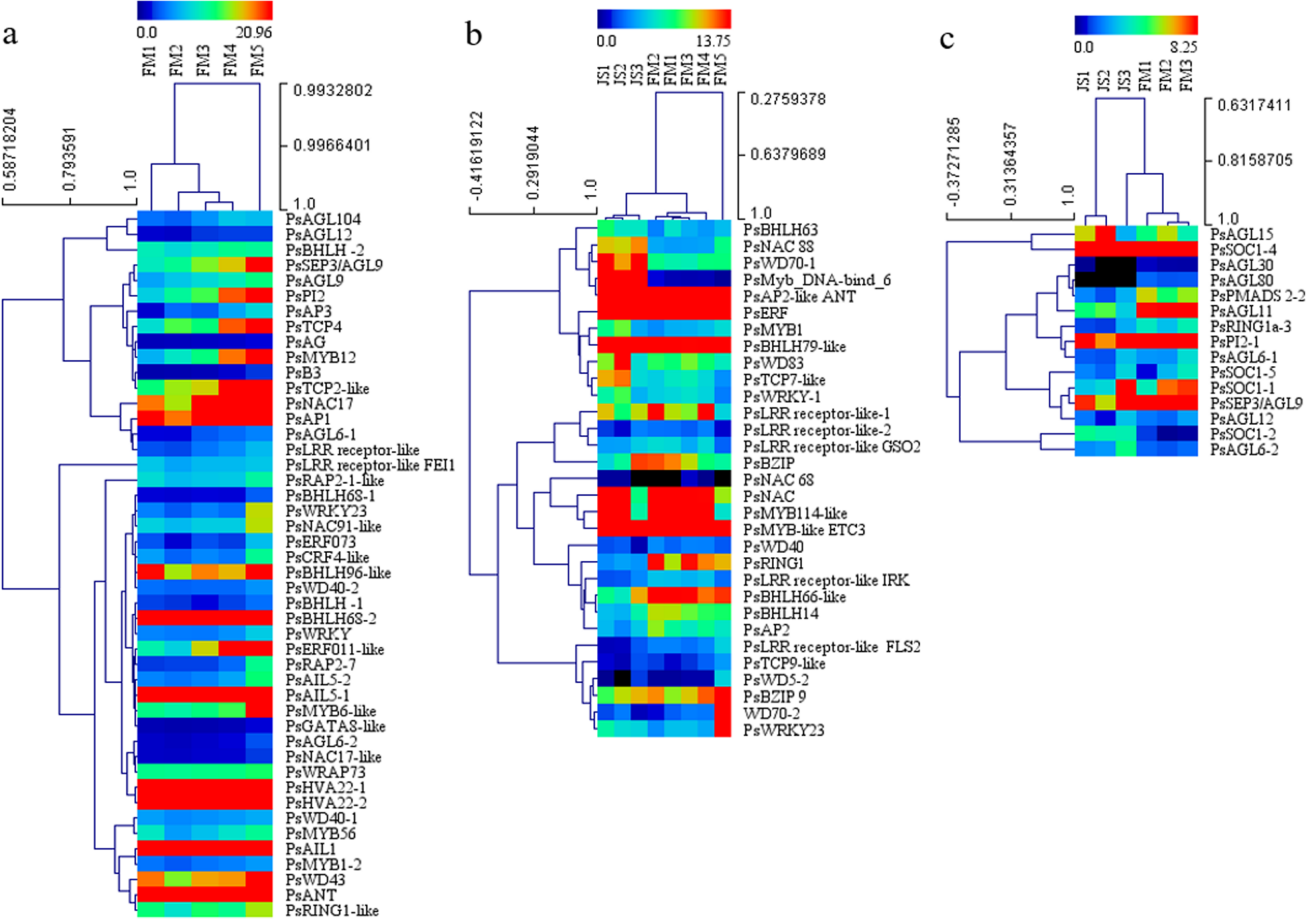
Heatmaps of DEGs associated with the carpel quantitative variation in *P. rockii*. **a** Heatmap of DEGs from profile 6, profile 14 and profile 41 in FM. **b** Heatmap of DEGs from JS and FM clustering pattern combinations. **c** Heatmap of DEGs from JS1–3 vs. FM1–3

All in all, we identified 46 DEGs associated with carpel quantitative variation in FM of *P. rockii*, including 10 MADS-box genes, 3 enzyme-like genes and 33 TFs from 10 gene families. Further, 17 DEGs with high expression levels were identified, which were annotated as *PsAP1*, *PsSEP3/AGL9*, *PsAGL9*, *PsPI2*, *PsANT*, *PsAIL1*, *PsAIL5–1*, *PsWD43*, *PsBHLH68–1*, *PsERF011-like*, *PsHVA22–1*, *PsHVA22–2*, *PsMYB12*, *PsMYB6-like*, *PsNAC17*, *PsTCP4* and *PsTCP2-like* (Fig. 4a).

### DEGs in JS vs. FM combinations

We identified 31 DEGs from 26 combinations of JS vs. FM, including 6 enzyme-like genes and 25 TFs from 7 gene families (AP2, NAC, BHLH, MYB, BZIP, WD and WRKY) (Fig. 3a, b and Additional file 9). Among them, the BHLH gene family showed the largest members, followed by the WD and MYB gene families. Further, 10 DEGs with high expression levels were identified, which were annotated as *PsAP2-like ANT*, *PsERF*, *PsBHLH79-like*, *PsBHLH66-like*, *PsBZIP9*, *PsMYB114-like*, *PsMYB-like ETC3*, *PsNAC*, *PsRING1* and *PsLRR receptor-like-1* (Fig. 4b). They could be considered as the candidate genes associated with carpel quantitative variation in *P. rockii*.

### DEGs in JS1–3 vs. FM1–3

We identified 11 AG-like DEGs in JS1–3 vs. FM 1–3, among which 6 DEGs were consistently upregulated (*PsAGL80*, *PsAGL30*, *PsAGL6–1*, *PsSEP3/AGL9*, *PsAGL12* and *PsAGL15*). Then 12 MADS-box DEGs were identified, including 6 consistently upregulated DEGs (*PsPI2–1*, *PsSOC1–1*, *PsSOC1–2*, *PsPMADS2–2*, *PsAGL11* and *PsSOC1–5*) and 2 consistently downregulated DEGs (*PsAGL6–2* and *PsSOC1–4*). We also identified 4 RING1-like DEGs, among which *PsRING1a-3* was consistently upregulated (Fig. 3c and Additional file 10).

In conclusion, 27 DEGs in JS1–3 vs. FM 1–3 associated with the carpel quantitative variation in *P. rockii* were identified. Among them, 13 DEGs were consistently upregulated and 2 DEGs were consistently downregulated. Further, we identified 7 DEGs with high expression levels, which were annotated as *PsSOC1–1*, *PsSOC1–4*, *PsAGL11*, *PsPMADS2–2*, *PsPI2–1*, *PsRING1a-3* and *PsSEP3/AGL9* (Fig. 4c).

### Phylogenetic analysis of DEGs

**Fig. 5:**
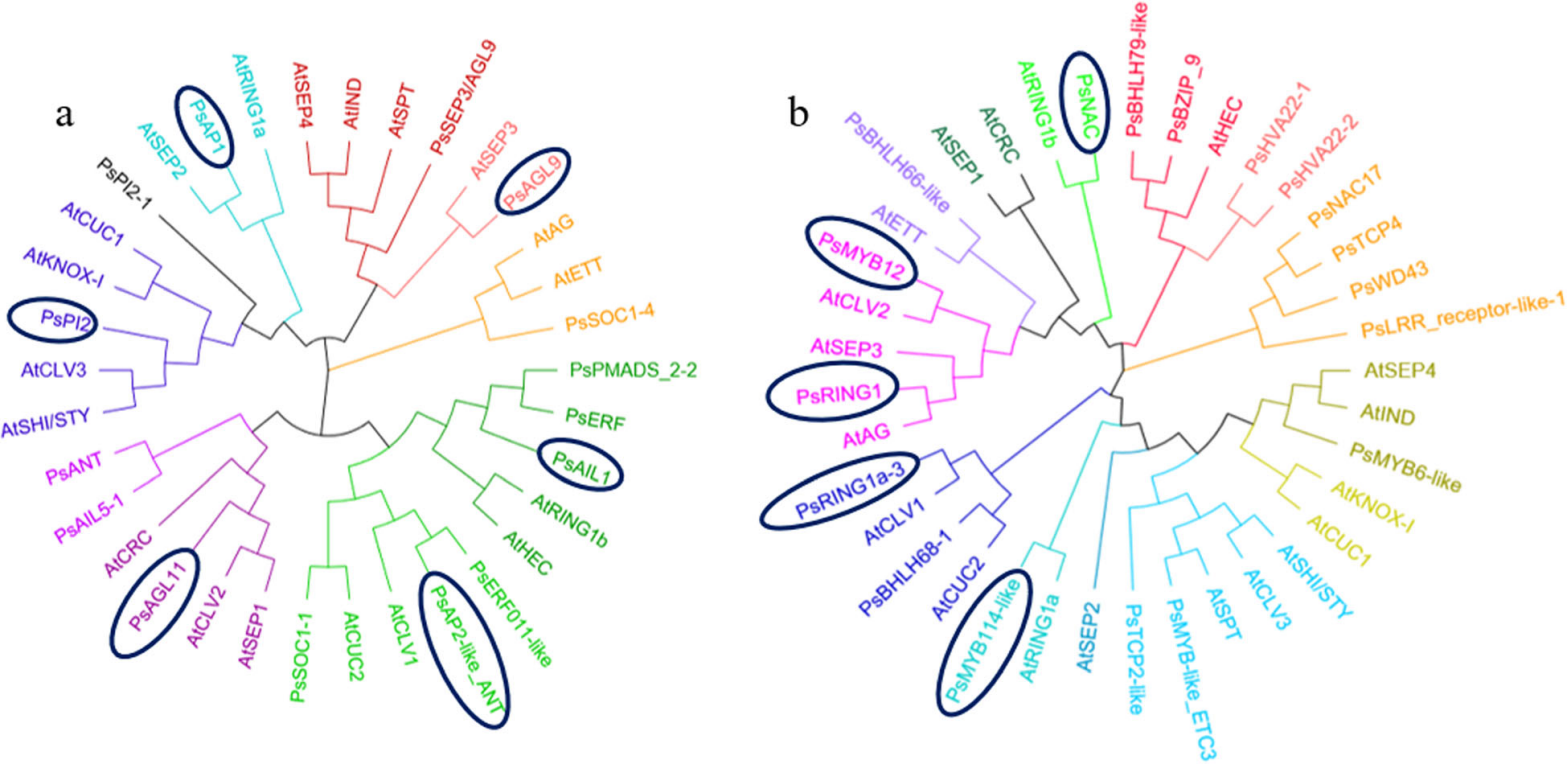
Phylogenetic analysis of DEGs. **a** The phylogenetic tree between floral organ development DEGs and the genes that regulate the carpel development and quantitative variation in *A. thaliana*. **b** The phylogenetic tree between the TFs, enzyme-like DEGs and the genes that regulate the carpel development and quantitative variation in *A. thaliana*. DEGs in the ellipse are highly homologous to the corresponding genes in *A. thaliana*. The amino acid sequences are derived from the NCBI database, and the phylogenetic trees are drawn using the MEGA7 software

Based on the above analysis, we identified 104 DEGs associated with carpel quantitative variation in *P. rockii*. Among them, 96 DEGs remained after removing duplicated genes. Then we identified 34 DEGs with high expression levels, including 16 floral organ development genes, 3 enzyme-like genes and 15 TFs. Further, we conducted phylogenetic analysis between the 34 DEGs and genes that regulated the carple development and quantitative variation in *A. thaliana*, as shown in Additional file 11. The results showed that the amino acid sequence similarity of DEGs in *P. rockii* was higher than that in *A. thaliana* (Fig. 5). We identified 6 DEGs with high homology to *A. thaliana* from 16 floral organ development candidate genes. Among them, *PsAGL11*, *PsPI2* and *PsAP2-like ANT* were homologous to the *AtCLV*-like genes, *PsAP1* and *PsAIL1* were homologous to the *AtRING1a/b*, and *PsAGL9* showed high homology with the *AtSEP3* (Fig. 5a). Then we identified 5 DEGs with high homology to *A. thaliana* from 15 TFs and 3 enzyme-like candidate genes. Among them, *PsMYB12* and *PsRING1a-3* were homologous to the *AtCLV*-like genes, *PsMYB114-like* and *PsNAC* were homologous to the *AtRING1a/b*, and *PsRING1* showed high homology with the *AtAG* (Fig. 5b).

All in all, we identified 11 candidate genes in *P. rockii* that were highly expressed levels and homologous to the genes in *A. thaliana*, including MADS-box (*PsAGL11*, *PsPI2*, *PsAP2-like ANT*, *PsAP1*, *PsAIL1* and *PsAGL9*), MYB (*PsMYB12* and *PsMYB114-like*), NAC (*PsNAC*) gene families and RING1 (*PsRING1a-3* and *PsRING1*) enzyme-like genes.

### Identification of gene coexpression modules

**Fig. 6:**
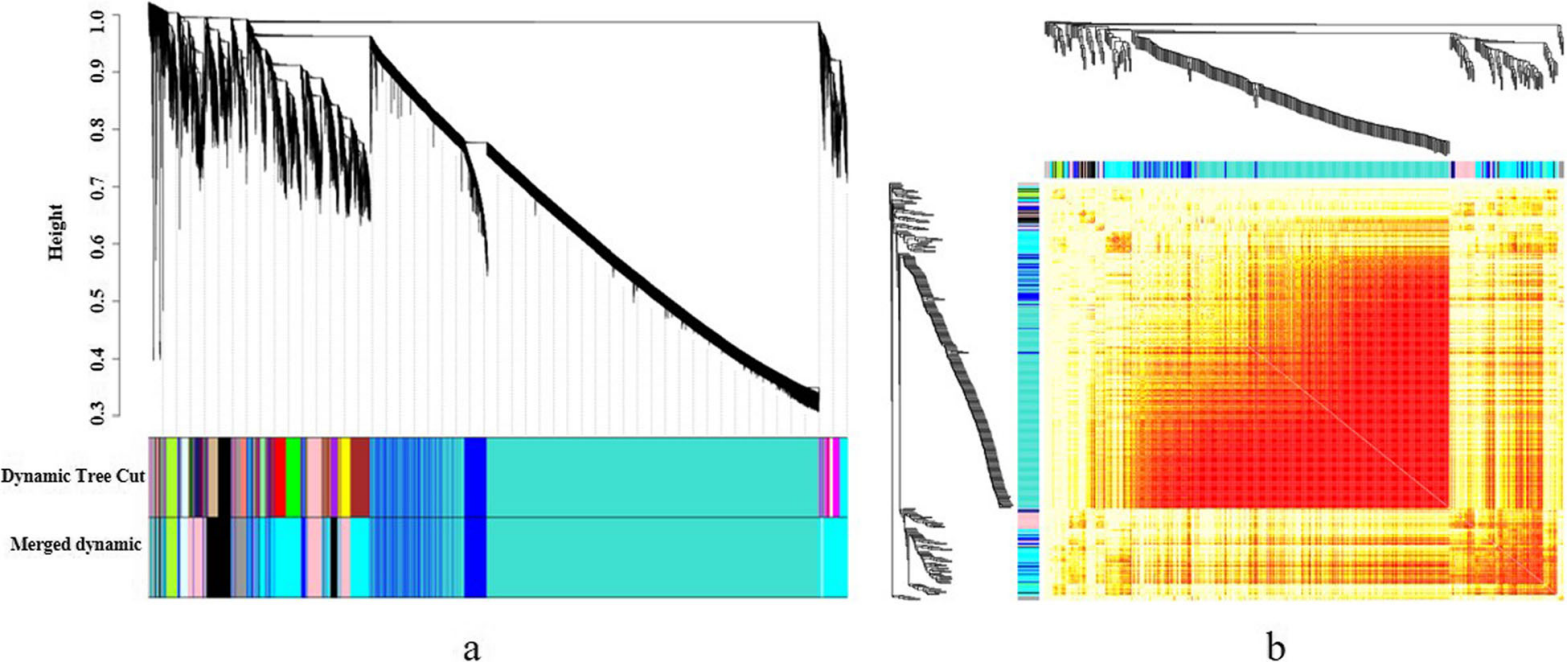
Weighted gene coexpression network in *P. rockii*. **a** Hierarchical clustering tree (dendrogram) of genes based on coexpression network analysis in JS and FM. Each ‘leaf’ (short vertical line) corresponds to individual gene. The branches correspond to modules of highly interconnected genes. Different colors below the dendrograms represent different gene modules and Merge corresponds to the result of combination of similar module. **b** The correlation coefficient heatmap of the coexpression module genes. Each bright spot corresponds to the correlation between each gene and other genes. The deeper the colors, the stronger is the connectivity between the two genes in the corresponding row and column

**Fig. 7:**
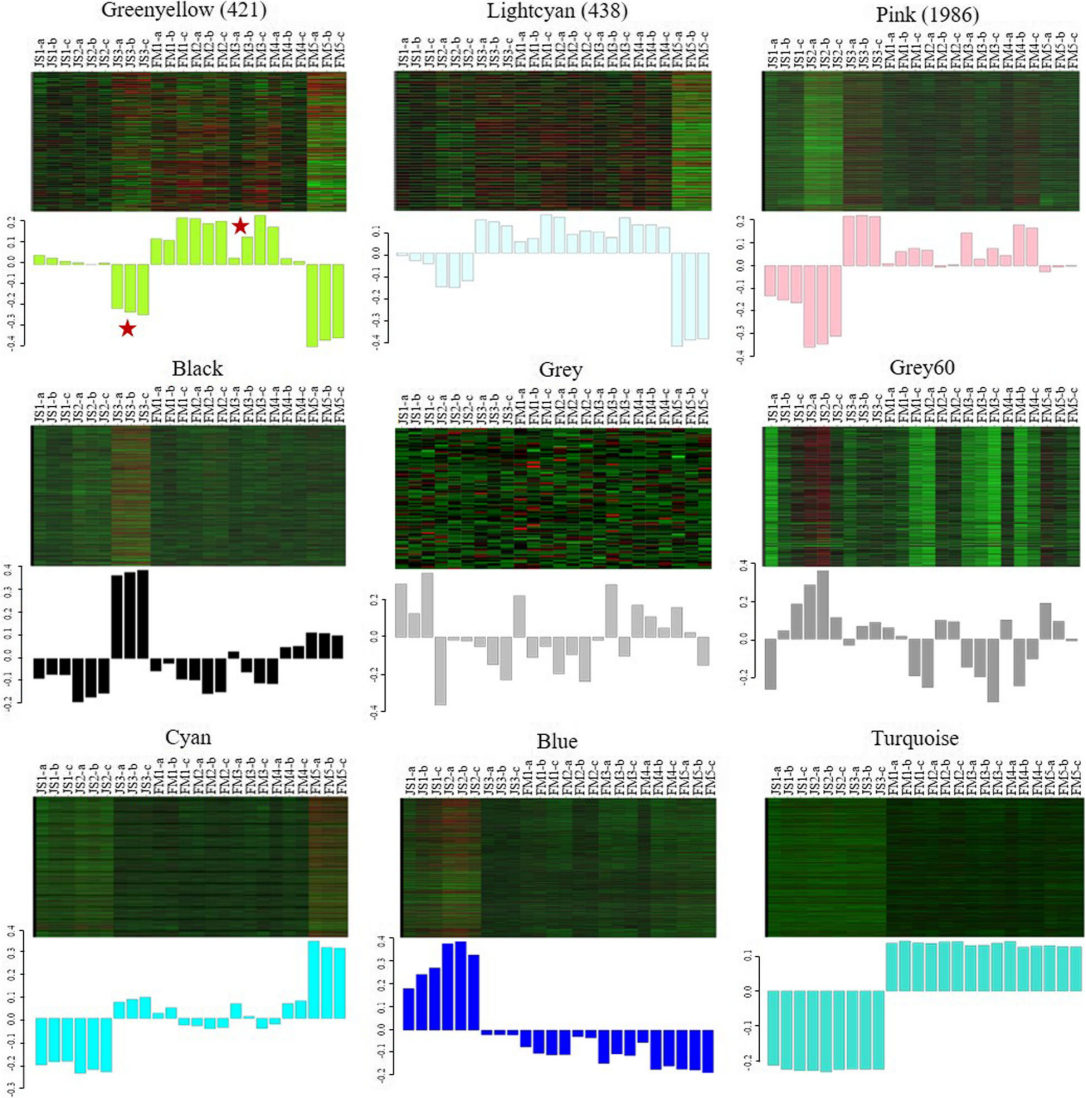
Coexpression modules via WGCNA. Heatmaps show the expression profiles of all the coexpressed genes in the modules (labeled on top). Bar graphs (below the heatmaps) show the consensus expression pattern of the coexpressed genes in each module. The number of coexpressed genes in the key coexpression modules are given on the top of the heatmaps. The bars showing the opposite expression patterns at JS3 and FM3 stages in the greenyellow module are marked with asterisks

**Fig. 8:**
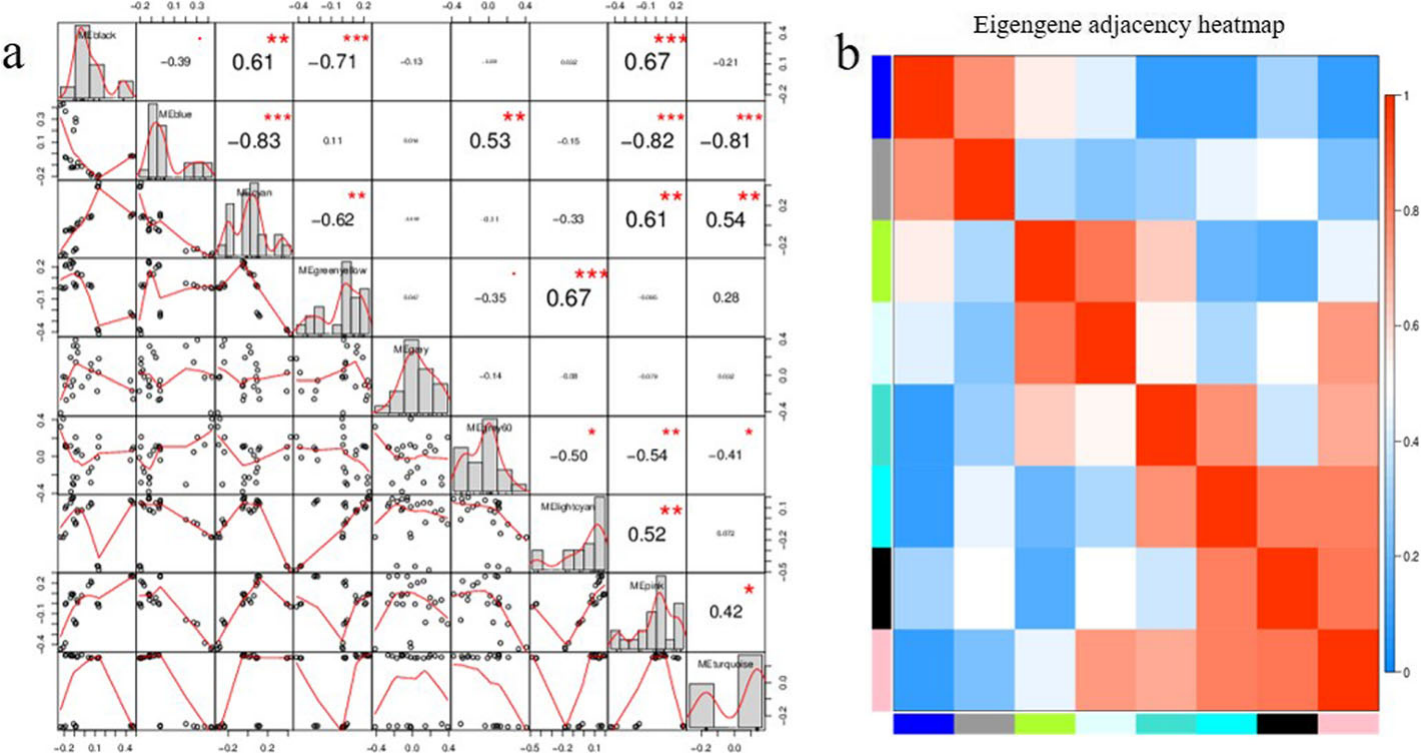
The correlation of coexpression modules. **a** The correlation diagram of coexpression modules. The diagonal corresponds to 9 coexpression modules. The lower left of the diagonal corresponds to the scatter diagrams between two modules. The number in the upper right of the diagonal represents the correlation value between two modules, and the asterisks indicate the degree of significance. **b** The clustering heatmap of coexpression modules

We first filtered the power values to make the gene distribution conform to the scale-free network. Then we observed that the power value (β value) was 9 when the correlation between k and p (k) was 0.85 and the average gene connectivity was 2000 (Additional file 12). Aimed to investigate the gene regulatory network associated with carpel quantitative variation in *P. rockii*, we identified coexpressed gene sets via WGCNA. Several major subnetworks representing interaction among genes with similar expression profiles were revealed, which were referred to as coexpression modules of DEGs (Fig. 6). Finally, a total of 9 modules were identified. We further screened three modules that were highly associated with carpel quantitative variation, including greenyellow, lightcyan and pink modules. Among them, the greenyellow module could be considered as the key module, as it showed noticeably the opposite expression trend at the key JS3 vs. FM3 stage (Fig. 7). In addition, we found that the greenyellow and lightcyan, lightcyan and pink modules all showed high correlations. And there were also correlations between greenyellow and black, pink and blue, pink and black modules, etc. (Fig. 8). Correlation analysis suggested that the DEGs in candidate modules might have similar functions.

### Coexpression network modules associated with the carpel quantitative variation in *P. rockii*

**Fig. 9:**
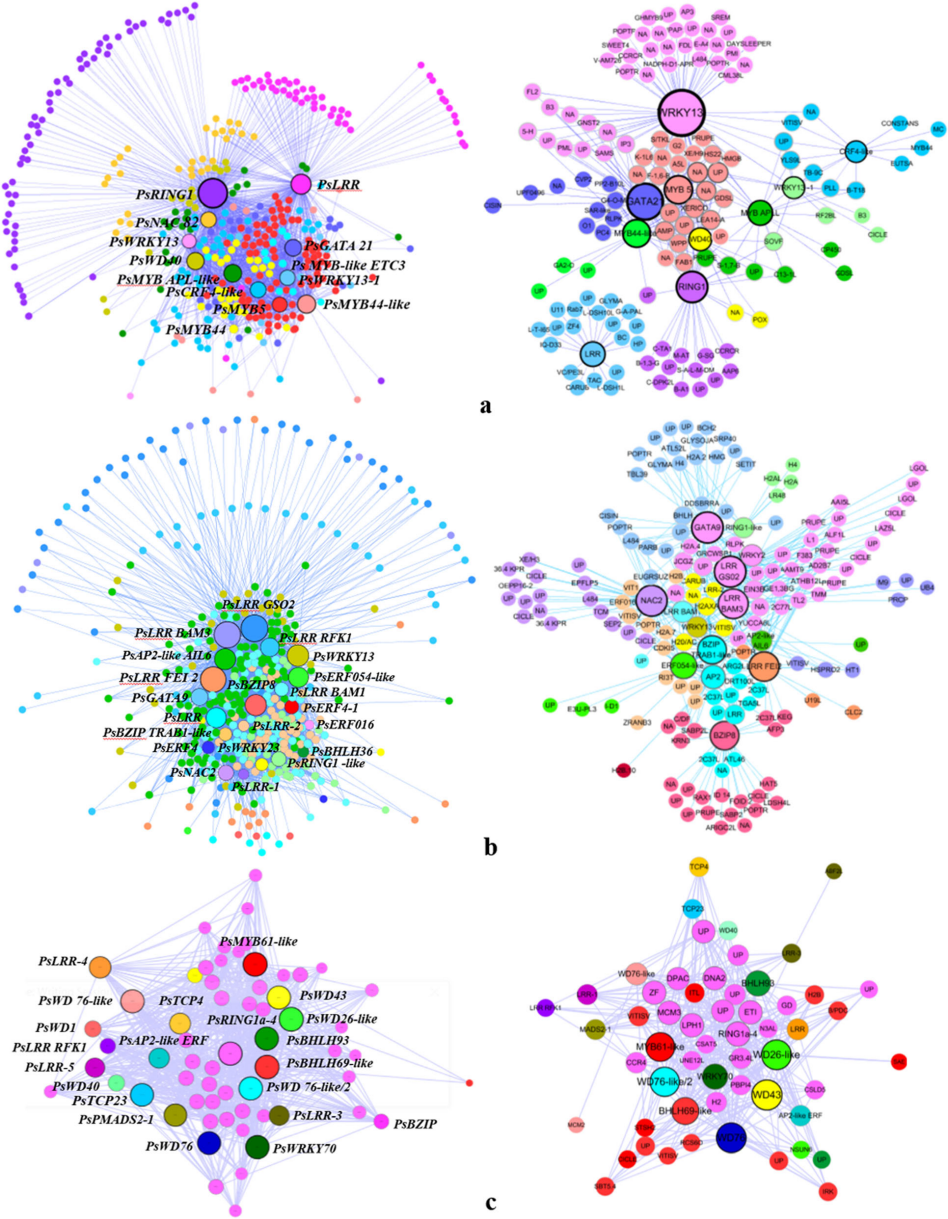
Transcriptional regulatory network associated with the carpel quantitative variation in *P. rockii*. **a**,**b**,**c** The transcriptional regulatory network of DEGs in greenyellow, lightcyan and pink modules. The size of the circles and words represents the interaction strength (sum of the correlation coefficients) between the genes. Different colors represent genes with different connectivity

We identified 1 floral organ developmental gene, 10 key TFs and 2 enzyme-like genes in the greenyellow module (Additional file 13). Using these DEGs as baits, we extracted the TOM values (relationship values between genes) of all the genes associated with them, and used Cytoscape 3.6.1 software to present the regulatory relationship between genes. The regulatory network showed that there was strong connectivity between the DEGs and other genes. The DEG with the strongest connectivity was PB.12928.2 (*PsRING1*), and the TF with the strongest connectivity was PB.48075.9 (*PsMYB44-like*). We observed that PB.44222.1 (*PsCRF4-like*), PB.65964.1 (*PsMYB-like ETC3*) and PB.54373.1 (*PsMYB114-like*) with strong connectivity were the same TFs displayed in the clustering pattern of DEGs. Further, we identified the coexpressed DEGs with TOM value greater than 0.1 and gene connection numbers greater than 5. As a result, *PsRING1*, *PsMYB44-like*, PB.53629.1(*PsWRKY13*), PB.54373.1(*MYB5*) and PB.51383.1(*GATA21*) showed strong connectivity (Fig. 9a).

We identified 5 floral organ developmental genes, 7 key TFs and 9 enzyme-like genes in the lightcyan module (Additional file 13). Then these DEGs were used as the baits to extract genes with connectivity, which were presented in the same method as the module described above. The DEG with the strongest connectivity was PB.14347.8 (*PsLRR BAM3*), and the TF with the strongest connectivity was PB.28248.1 (*PsWRKY 13*). Moreover, PB.6199.16 (*PsLRR like-1*), PB.9500.9 (*PsLRR GSO2*) and PB.60580.1 (*PsWRKY23*) with strong connectivity were the same TFs displayed in the clustering pattern of DEGs. Further, the coexpressed DEGs with the same screening conditions as the above module were identified. Finally, 8 DEGs showed strong connectivity, including *PsLRR-like BAM3*, *PsLRR GSO2*, PB.54070.1(*PsNCA2*), PB.40019.1(*PsERF054-like*), PB.35331.4 (*PsBZIP8*), PB.52258.1 (*PsGATA 9*), PB.2902.1 (*PsLRR FEI2*) and PB.60155.1 (*PsBZIP TRAB1-like*) (Fig. 9b).

We identified 2 floral organ developmental genes, 14 key TFs and 5 enzyme-like genes in the pink module (Additional file 13). Then these DEGs were used as the baits to extract genes with connectivity, which were presented in the same method as the modules described above. The DEGs with the strongest connectivity included PB.28178.3 (*PsWD 26-like*), PB.48069.2 (*PsMYB61-like*) and PB.48928.1 (*PsBHLH69-like*). Moreover, PB.62468.1 (*PsPMADS 2–1*), PB.1586.14 (*PsLRR -2*), PB.48801.1(*PsRING1a-4*), PB.52867.3 (*PsBZIP*) with strong connectivity were the same DEGs displayed in the clustering pattern. Further, the coexpressed DEGs with the same screening conditions as the above modules were identified. Finally, 8 DEGs showed strong connectivity, including *PsBHLH69-like*, *PsMYB61-like*, PB.34757.12 (*PsWD43*), PB.48506.7 (*PsBHLH93*), PB.56783.1(*PsWRKY70*), PB.31962.6 (*PsWD76-like/2*), PB.9989.2 (*PsWD76*) and PB.28178.3 (*PsWD26-like*) showed strong connectivity (Fig. 9c).

**Fig. 10:**
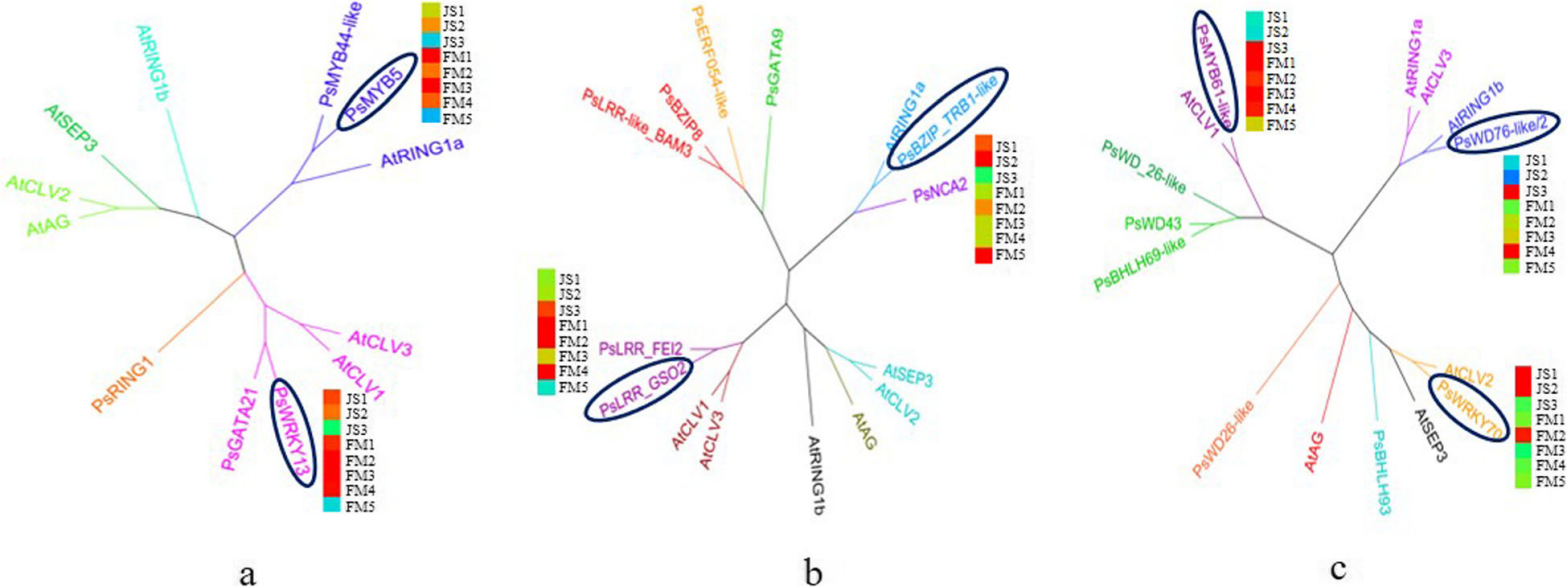
Phylogenetic analysis of DEGs in coexpression modules. **a**, **b**, **c** The phylogenetic tree of DEGs in the greenyellow, lightcyan and pink modules. DEGs in the ellipse are highly homologous to the genes that regulate the carpel development and quantitative variation in *A. thaliana*. The heatmaps correspond to the expression levels of DEGs at JS1–3 and FM1–5 stages. The amino acid sequences of the genes in *A. thaliana* are derived from the NCBI database, and the phylogenetic trees are drawn using the MEGA7 software

All in all, a total of 21 DEGs with strong connectivity in the greenyellow, lightcyan and pink modules were identified in *P. rockii*. Then phylogenetic analysis revealed that *PsWRKY13*, *PsLRR GSO2*, *PsMYB61-like* and *PsWRKY70* showed high homology with the *AtCLV*-like genes. *PsMYB5*, *PsBZIP TRB1-like* and *PsWD76-like/2* were homologous to the *AtRING1a/b*. Among them, the DEGs with high expression levels in the critical stages included *PsWRKY13*, *PsLRR GSO2*, *PsMYB61-like* and *PsWD76-like/2* (Fig. 10). Therefore, we finally identified 4 candidate genes that showed strong connectivity, high expression levels and high homology with the genes in *A. thaliana* by WGCNA.

### Gene expression validation

**Fig. 11:**
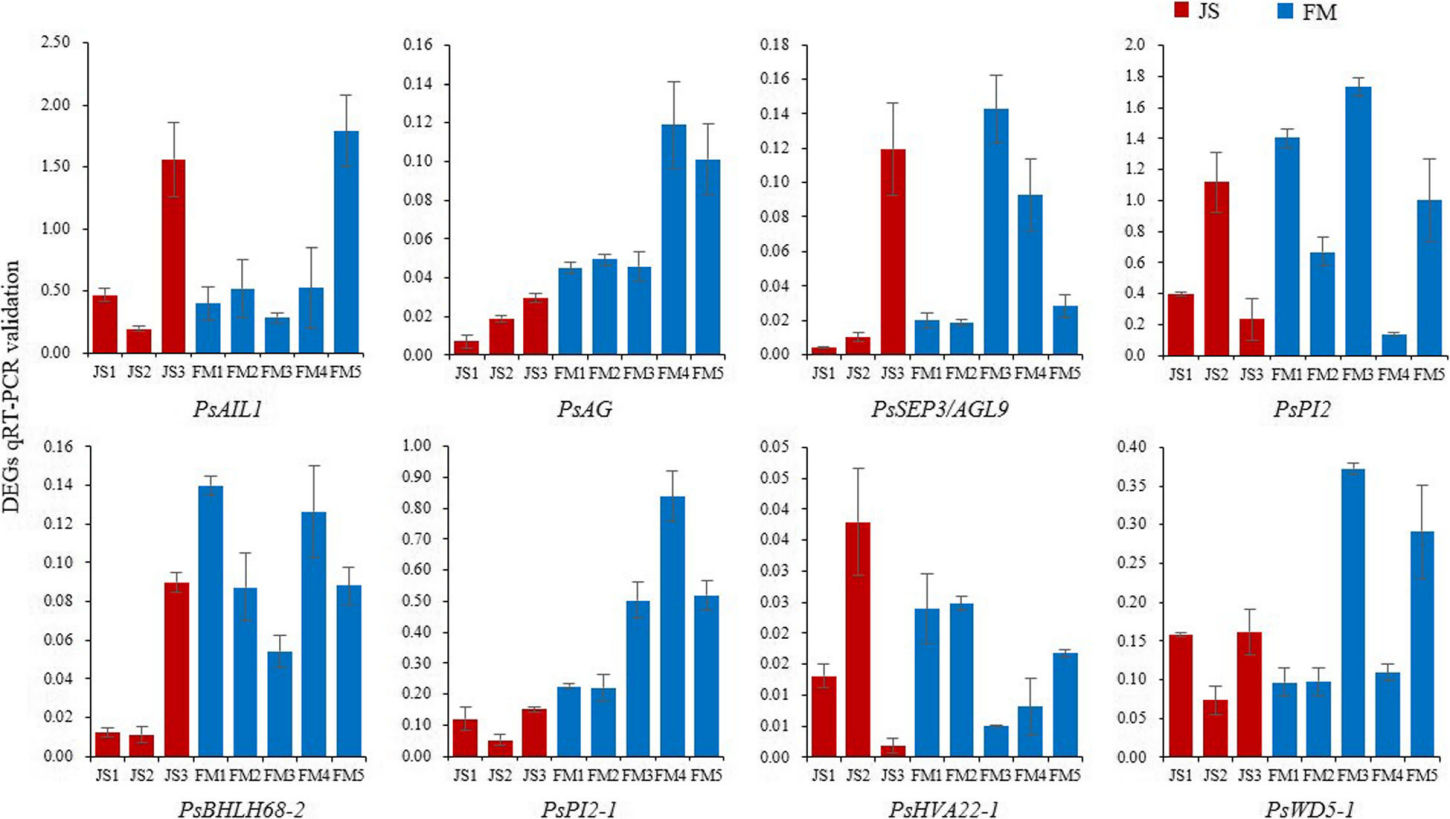
The qRT-PCR validation results of 8 DEGs in the transcriptome. Red bars show the expression of JS1–3. Blue bars show the expression of FM1–5

A scale of DEGs associated with the carpel quantitative variation was selected to test the expression profiles by the real-time quantitative polymerase chain reaction (qRT-PCR) analysis. We randomly selected eight DEGs in the transcriptome and designed specific primers (Fig. 11 and Additional file 14). As a result, the DEGs were upregulated in FM3–4/FM3–5, or showed different expression trends at key sampling stages in JS vs. FM, or were consistently upregulated/downregulated in JS1–3 vs. FM1–3. These results were primarily coincident with those shown by RNA-Seq in expression tendency and demonstrated the credibility of sequencing data and the pattern profiles. These DEGs were reference candidate genes that synergistically regulated the carpel quantitative variation in *P. rockii*.

## Discussion

### Study on carpel quantitative variation in *P. rockii* by combining two generations of RNA-seq

Because there have been no genomic information of *Paeonia* yet, RNA-seq technology has become an important means of screening candidate genes associated with key traits [40, 42]. However, huge genomic information (about 12.5 G) and complex genetic background make it very difficult to identify gene loci effectively by the NGS method only. Then this study is the first attempt to combine two generations of RNA-seq methods to explore the carpel quantitative variation in *Paeonia*. Compared with the previous reports concerned about tree peonies, we present a higher quality sequencing products [50–54]. For example, more than 60% of the sequence reads are 1000~3500 bp, and the longest ones are over 5000 bp. This is a relatively high proportion of the long-read transcript sequences currently obtained from *P. rockii*. Therefore, we confirme that the efficient application of RNA-seq provides more comprehensive resources for the study of floral organ development in tree peonies.

### Functional loci of carpel quantitative variation in *P. rockii*

Studies have shown that the development and quantitative variation of carpel are closely related to the floral organ development factors [15–22]. In this study, we finally identified 6 floral development DEGs (*PsAGL11*, *PsPI2*, *PsAP2-like ANT*, *PsAP1*, *PsAIL1*, *PsAGL9*) associated with carpel quantitative variation in *P. rockii*. This is consistent with the results reported in *A. thaliana* (D-class and AG-SEP3) [15–17], *O. sativa* (*MFO1*/*MADS6*) [18], *Z. mays* (*bde* and *zag1*) [19–21] and *V. vinifera* [22]. Then we speculate that these DEGs may have similar function in regulating carpel quantitative variation as in other crops. Moreover, CLV-like genes have been shown to be important regulators of carpel quantitative variation [23–27, 30, 31]. However, we did not directly screen the CLV-like genes in the DEGs profiles. We speculate that this may be the result of differences in the sampling stages. In CLV-like genes plants, the apical meristem can fasciate in the more severe mutant alleles, and this fasciation can occur prior to the transition to flowering. But the materials collected in this study were mainly at the stages of stamen and carpel primordia. Nevertheless, we identified DEGs that showed high homology with the genes regulating the number of carpels in *A. thaliana*. The function of the DEGs remains to be further verified in further studies.

### WGCNA of carpel quantitative variation in *P. rockii*

Clustering pattern analysis of gene expression is commonly used in transcriptome research [34–36]. However, we found that some DEGs identified by clustering pattern in this study were differentially expressed in both cultivars of *P. rockii*, which limited the identification of specific factors. In contrast, WGCNA can effectively screen information through specific modules and intergene connectivity [44–49].

In this study, we observed that all three key modules contained the same DEGs as in the clustering patterns, such as *PsMYB114-like*, *PsLRR like-1*, *PsPMADS 2–1*, etc. The results indicate that there are certain similarities between the key clustering patterns and coexpression modules. Studies have shown that WD40, RING1 and LRR gene families are widespread in plants and involved in signal transduction and gene transcription regulation, etc. [55–57]. In *A. thaliana*, AtRING1 is the core component of PRC1 and regulates stem cell-determining carpel development [28]. In *P. rockii*, RING1-like TFs showed strong connectivity in the coexpression network relationship of carpel quantitative variation. These results indicate that RING1-like genes are closely related to the carpel development, and we speculate that RING1-like DEGs are probably upstream factors. Additionally, many WD40 homologues in *A. thaliana* regulate the carpel development and variation, for example the CLV1, CLV2, CLV3 and WUS in *A. thaliana* are modulated by AaWD40 and Arabidopsis TTG1, and CYP71 deletion mutant *cyp71* can increase the number of carpel [58–60]. In this study, we observed that WD-like TFs showed high activity and strong connectivity in carpel quantitative variation, among which *PsWD76-like/2* showed high homology with *AtRING1b*. Therefore, we speculate that WD-like DEGs have a close upstream regulatory relationship with WUS and CLV-like genes particularly in *P. rockii.* Besides, LRR-RLKs participate in the signaling networks that regulate stem cell development by sensing CLV3 polypeptide and inhibiting the expression of WUS gene in rib meristem in *A. thaliana* [61, 62]. In this study, LRR-like DEGs show relatively stronger connectivity than other genes. We speculate that they are likely to be the important regulators in upstream regulatory networks of carpel quantitative variation.

### MYB-like TFs of the carpel quantitative variation in *P. rockii*

The MYB family of proteins is large, functionally diverse and represent in all eukaryotes, and MYB proteins are the key factors in regulatory networks controlling development, metabolism, etc. [63–66]. Studies have also shown that the MYB gene family is associated with carpel development [67–71]. In this study, we identified 3 MYB-like TFs associated with the carpel quantitative variation, including *PsMYB114-like*, *PsMYB12* and *PsMYB61-like*. Among them, *PsMYB114-like* showed strong connectivity and high homology with *AtRING1a*, which negatively regulated *KNOX-1* expression in *A. thaliana* [28]. And *AtMYB91*/*AS1* has also been shown to negatively regulate *KNOX* (*KNOTTED*) expression in organ primordia [69]. Therefore, we further conducted phylogenetic analysis and found that *PsMYB114-like* also showed high homology with *AtMYB91*/*AS1* (Additional file 15). Then we speculate that *PsMYB114-like* is very likely to negatively regulated *KNOX* expression in the WUS-*KNOX* pathway of carpel quantitative variation. Additionally, we observed that MYB was the only candidate gene family identified in both clustering pattern and WGCNA. *PsMYB12* and *PsMYB61-like* respectively show strong connectivity and high homology with *AtCLV2* and *AtCLV1*. We speculate that they may be the key regulatory factors in the CLV-WUS pathway of carpel quantitative variation in *P. rockii* [28].

### Hypothetical model of the regulatory network for carpel quantitative variation in *P. rockii*

**Fig. 12:**
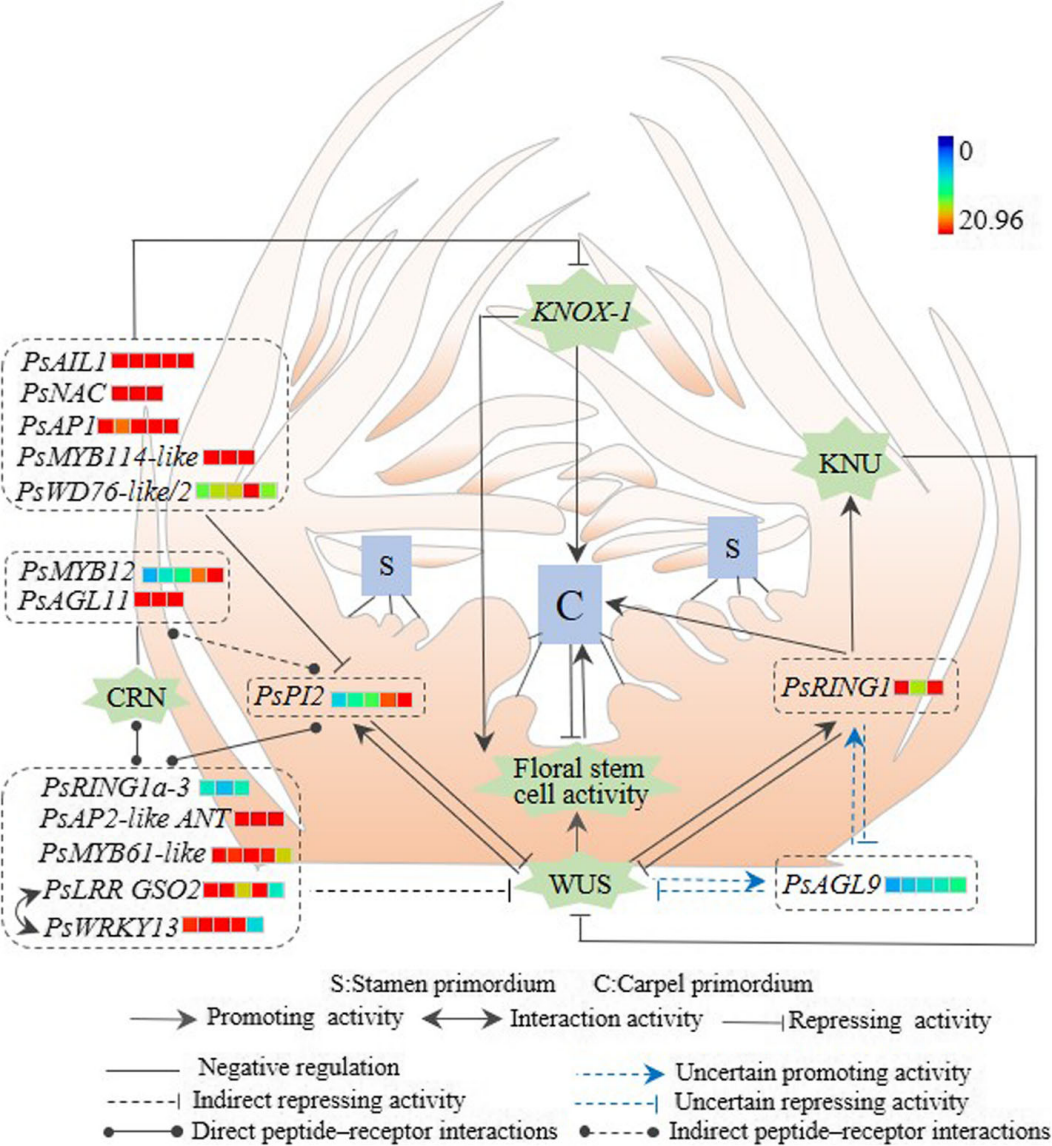
Hypothetical model of the regulatory networks for the carpel quantitative variation in *P. rockii*. Heatmaps correspond to the expression levels of candidate genes in FM1–5 or FM1–3

The CLV-WUS stem cell signaling pathway, *KNOX-I*, *CLV3* and the *WUS*-*AG*-*KNU* feedback loop are indicated in *A. thaliana* [26, 28], on which a hypothetical model of the regulatory network for the carpel quantitative variation in *P. rockii* is proposed, as shown in Fig. 12. First, proper carpel initiation depends on the WUS-AG pathway. *PsRING1* and *PsAGL9* are homologous to *AtAG* and *AtSEP3*, respectively. We speculate that *PsAGL9* is indirectly involved in the WUS-AG pathway in *P. rockii*. Moreover, it has also been well established that CLV3 regulats carpel development through AG [72, 73]. Then we speculate that *PsPI2* (homologous to *AtCLV3*) also works in CLV-AG pathway in this study. Further, CLV3 peptides can be bound by the CLV1 LRR-RLK in the CLV-WUS pathway and they function in the same pathway to regulate meristem development [25, 26]. Then we speculate that there are also direct peptide-receptor interactions between *PsRING1a-3*, *PsAP2-like ANT*, *PsMYB61-like*, *PsLRR GSO2*, *PsWRKY13* and *PsPI2*. Among them, we confirm the interaction between *PsLRR GSO2* and *PsWRKY13* through WGCNA, while the regulatory relationship between other genes need to be further validated. In addition, the CLV2 LRR receptor-like protein and the CORYNE (CRN) protein are the second distinct receptor complex of CLV3 in the CLV-WUS pathway [26]. Therefore, there are indirect peptide-receptor interactions between *PsMYB12*, *PsAGL11* and *PsPI2*. Finally, *PsAIL1*, *PsNAC*, *PsAP1*, *PsMYB114-like* and *PsWD76-like/2* show high homology with *AtRING1a/b*. We speculate they may regulate stem cell-determining carpel development mainly through repression of class I *KNOX* genes in *P. rockii* [28]. Taken together, the regulatory networks between candidate genes still need to be further verified in *P. rockii*.

## Conclusions

In comparative transcriptome, vital differences of gene expression between FM and JS were observed in this study. The floral organ development genes (*PsAGL11*, *PsPI2*, *PsAP2-like ANT*, *PsAP1*, *PsAIL1*, *PsAGL9*), TFs (*PsMYB114-like*, *PsMYB12*, *PsMYB61-like*, *PsNAC*, *PsWRKY13*, *PsWD76-like/2*) and enzyme-like genes (*PsRING1*, *PsRING1a-3*, *PsLRR GSO2*) at critical stages were identified, which provided a distinct view of the molecular mechanism of carpel quantitative variation in *P. rockii*. Moreover, the NGS and SMLRS techniques were helpful to discover the key TFs and enzyme-like genes. Among them, floral organ development gen es were the key factors, and *PsMYBs*, *PsWDs*, *PsRING1*s and *PsLRRs* might be the upstream factors to regulate the carpel quantitative variation in *P. rockii*.

All these results furnish practical information to explore the carpel quantitative variation in tree peonies. Meanwhile, since carpel development is closely associated with seed formation and the yield, it is also of great significance to the selection of yield traits and the cultivation of high yield cultivars for oil tree peony. Perhaps the more interesting questions arising from this research are the identification of exact genes contributing to the development and quantitative variation of carpels, and how those genes are regulated by the TFs. In-depth studies are required to answer these questions and to provide an insight into the regulatory network and induction mechanism of carpel quantitative variation in woody plants including tree peonies.

## Methods

### Plant materials collection

The experimental materials were obtained from the terminal and axillary flower buds of two *P. rockii* cultivars (JS and FM), which were collected from living adult plants grown in Beijing Guose Peony Garden at Beijing, China (40°45′N, 115°97′E). At each stage of material collection, 3–5 flower buds of similar size were collected from 9:00 to 11:00 every day for FAA fixation (38% formaldehyde 5 ml; glacial acetic acid 5 ml; 50% ethyl alcohol 90 ml). Then samples were observed under an anatomical microscope to identify the stages of flower buds differentiation. The samples were collected at the stages of JS1–3 in JS and FM1–5 in FM, as shown in Fig. 1. Approximately 10 flower buds of similar size in the same node were collected as a biological replicate at each stage, and three biological replicates were collected. We measured the length and diameter of each flower bud using a vernier caliper. The samples were frozen in liquid nitrogen immediately after removing the scales and part of the leaf primordia and then stored at − 80 °C for extraction of total RNA.

### RNA extraction, database construction, sequel and illumina sequencing

Total RNA was extracted from the flower buds of JS and FM cultivars applying the EASYspin Plus Complex Plant RNA kit (Aidlab Biotechnologirs Co., Ltd. Beijing, China) according to the manufacturer’s instructions. Then, 5 μL of each total RNA sample was assessed by 1% Tris-acetic acid-EDTA (TAE) agarose gel electrophoresis. Simultaneously, 1 μL of each total RNA sample was assessed using a NanoDrop ND2000, A260/A280 > 2.0, 28S/18S > 1.0, and RNA concentration ≥ 500 ng/μL [74]. The library was constructed after the samples passed quality inspection. The cDNA library was constructed by magnetic bead enrichment and PCR amplification in the NGS method. The SMLRS method constructed 8 libraries applying Sequel; 1~2 K library detected 2 cells, 2~3 K library detected 2 cells and > 3 K library detected 1 cell. After the completion of the database, the NGS method used the Illumina HiSeq Xten platform to perform paired-end sequencing on the constructed cDNA library, with 150 PE and 6 G raw data per sample. The SMLRS method used reagent V2 to transfer the library template and enzyme complex into the nanopore of the PacBio Sequel sequencer for real-time RNA-seq, and a total of 3 SMRT cells were detected (Nextomics Biosciences Co., Ltd. Wuhan, China).

### DEGs selection, function annotation, clustering pattern and WGCNA

For the assembly library of NGS method, raw data in fastq format was first processed using in-house Perl scripts. The raw reads were filtered by removing adapter, reads containing poly-N and low-quality sequences. Then clean reads were de novo assembled using Trinity [75], and the transcriptome reference database was obtained. For the SMLRS method, the PacBio Sequel sequencing data was saved in BAM format, and post-filter polymerase reads were obtained by filtering connectors and removing low-quality sequences. The filter criteria for reads of insert was a minimum full passes of 1 and a minimum prediction accuracy of 0.80, then reads of insert were classified and full-length reads were obtained. Further we used the ICE algorithm module in the ICE package for cluster analysis, and got the consistency sequence through DAGCon. After correction by Quiver, isoforms were obtained (parameter: -c, 0.99; −T, 6; −G, 0; −aL, 0.90; −AL, 100; −aS, 0.99; −AS, 30). Finally, unigenes were obtained through the CD-HIT cluster analysis (parameter: -T, 12; −M, 45000; −c, 0.85).

In this study, the RPKM method and RSEM software (v1.1.12) were used to calculate the expression levels of genes [76]. Analysis of DEGs was performed using EdgeR software. The *P*-value of difference test was corrected by multiple hypothesis test, and the domain value of the *P*-value was determined by controlling the FDR (false discovery rate) [77]. *P*-value ≤0.05 and FDR ≤ 0.01 were taken as the thresholds for screening DEGs. Blastx was used to annotate the function of unigenes, which were referenced to the Nr, Swiss-Port, Kyoto Encyclopedia of Genes and Genomes (KEGG) and Cluster of Orthologous Groups of proteins (COG) databases (*E*-value< 0.00001). The STEM (Short Time series Expression Miner) software (v1.3.11) (http://www.cs.cmu.edu/~jernst/stem/) was used to analyze the clustering patterns of DEGs, then functional enrichment analysis was performed for all clustering patterns.

Additionally, the DEGs were enriched using the R language package and the coexpression network was constructed by the WGCNA algorithm. Based on log_2_ (1 + FPKM) values, a matrix of pairwise SCCs between all pairs of genes was generated and transformed into an adjacency matrix using the formula: connection strength (adjacency value) = |(1 + correlation)/2| Power. Here, Power represents soft threshold for the correlation matrix. A Power value of 9 was selected based on the scale-free topology criterion. The resulting adjacency matrix was converted to a topological overlap (TO) matrix via TOM similarity algorithm, and the genes were hierarchically clustered based on TO similarity. The dynamic tree-cutting algorithm was used to cut the hierarchal clustering dendrogram and modules were defined after decomposing/combining branches to reach a stable number of clusters [78]. For each module, a summary profile was calculated via PCA. The regulatory relationship between DEGs in the modules was represented by Cytoscape 3.6.1 software (http://www.cytoscape.org/download.php).

### qRT-PCR verification

The DEGs screened according to the analysis results were verified by qRT-PCR. The template was the firststrand cDNA synthesized from 1 μg total RNA using a TUREscript cDNA Synthesize Kit (Aidlab Biotechnologirs Co., Ltd. Beijing, China) according to the manufacturer’s instructions. The fluorescent dye was SYBR Green PCR Master Mix (TaKaRa, Japan). The specific primers were designed with Primer Primer 5.0 software (Additional file 14). Expression levels were normalized against the reference gene *UBIQUITIN* [79]. The reactions were carried out in a 20 μL volume containing 10 μL SYBR Green PCR Master Mix, 0.4 μL each primer, 7.2 μL dd H_2_O, 2 μL template cDNA under the following conditions: 30 s at 95 °C, 40 cycles of 5 s at 95 °C, 30 s at 55 °C and 30 s at 72 °C. Realtime RT-PCR was conducted using the qTOWER2.2 PCR System (Jena, Germany). Three biological replicates and technical replicates were performed in all qRT-PCR experiments, and the expression levels of candidate genes was calculated using the 2^-ΔΔCt^ method.

## Additional files

Additional file 1: Throughput and quality of next-generation and single-molecule long-read RNA-seq.(XLSX 12 kb)

Additional file 2: Venn diagram of number of unigenes annotated by BLAXTx against protein databases.(JPG 104 kb)

Additional file 3: The expression distribution of all unigenes obtained by RNA-seq.(JPG 125 kb)

Additional file 4: The correlation heatmap of all samples in JS and FM cultivar.(JPG 123 kb)

Additional file 5: GO term and KEGG pathway enrichment statistics of DEGs in profile 41 of FM cultivar.(JPG 239 kb)

Additional file 6: The DEGs of significant enrichment patterns (profile 6, profile14 and profile 41) in FM cultivar.(XLSX 12 kb)

Additional file 7: GO term and KEGG pathway enrichment statistics of DEGs in profile 14 of FM cultivar.(JPG 138 kb)

Additional file 8: GO term and KEGG pathway enrichment statistics of DEGs in profile 6 of FM cultivar.(JPG 248 kb)

Additional file 9: The DEGs in JS vs. FM combinations.(XLSX 11 kb)

Additional file 10: The DEGs in JS1-3 vs. FM1-3.(XLSX 11 kb)

Additional file 11: The genes that regulate the development and quantitative variation of carpels in *A*. *thaliana*.(XLSX 10 kb)

Additional file 12: Filtering of power value for gene network weight analysis.(JPG 54 kb)

Additional file 13: The DEGs in greenyellow, lightcyan and pink modules.(XLSX 12 kb)

Additional file 14: Primers for qRT-PCR analysis.(XLSX 10 kb)

Additional file 15: Phylogenetic analysis of MYB-like DEGs in *P. rockii* and *AtMYB91*/*AS1* in *A. thaliana.*(JPG 95 kb)

## Data Availability

The sequence raw data from this study have been submitted to the NCBI Sequence Read Archive (SRR9915032) (https://www.ncbi.nlm.nih.gov/sra/?term=SRR9915032).

## Abbreviations

*A. thaliana*: *Arabidopsis thaliana*
DEG: Differentially expressed gene
FM: *P. rockii* ‘Fenmiantaosai’
FM4: The second whorl of carpel primordia initial appearance stage
FM5: The second whorl of carpel primordia ventral suture formation stage
GO: Gene ontology
JS: *P. rockii* ‘Jingshunfen’
JS1, FM1: The stamen primordium stage
JS2, FM2: The first whorl of carpel primordia initial appearance stage
JS3, FM3: The first whorl of carpel primordia ventral suture formation stage
KEGG: Kyoto encyclopedia of genes and genomes
NCBI: National Center for Biotechnology Information
NGS: Next-generation sequencing
*O. sativa*: *Oryza sativa*
*P. rockii*: *Paeonia rockii*
PCR: Polymerase chain reaction
SMLRS: Single-molecule long-read sequencing
TF: Transcription factor
WGCNA: Weighted gene coexpression network analysis
*Z. mays*: *Zea mays*
