# Supplementary figures and images for: Comparative transcriptome and coexpression network analysis of carpel quantitative variation in Paeonia rockii
Source: BMC Genomics. 2019 Aug 29;20:683. doi: 10.1186/s12864-019-6036-z (PMC6716868; doi:10.1186/s12864-019-6036-z)

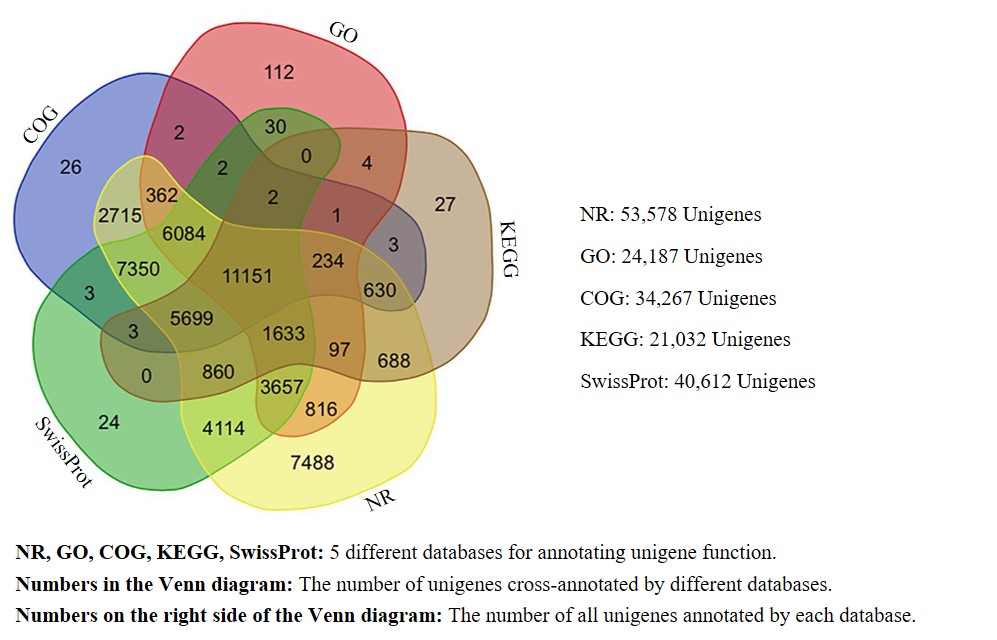

Supplement: Supplementary file 2 — Venn diagram of number of unigenes annotated by BLAXTx against protein databases.(JPG 104 kb) [file 12864_2019_6036_MOESM2_ESM.jpg]

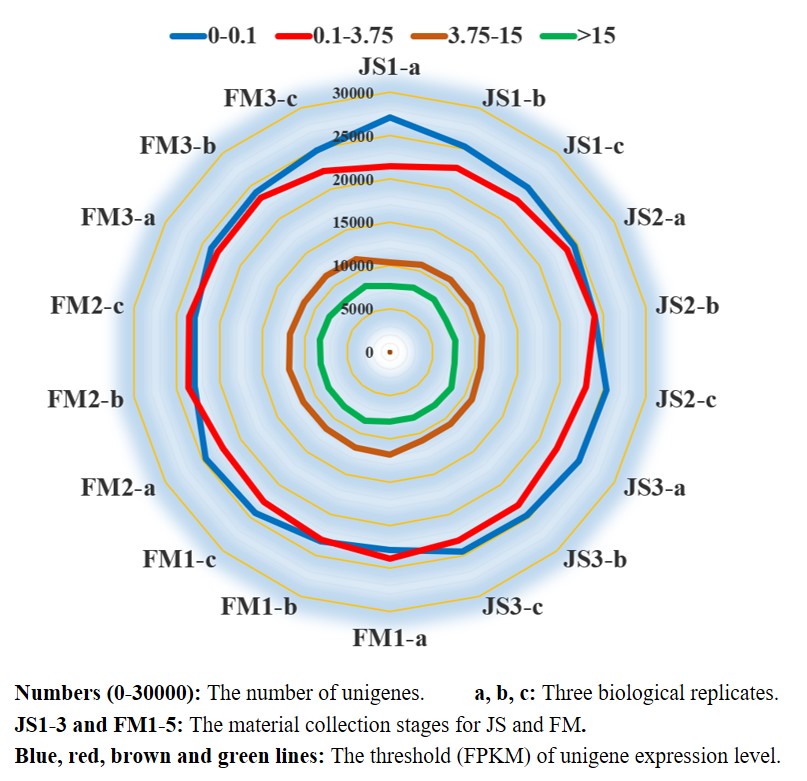

Supplement: Supplementary file 3 — The expression distribution of all unigenes obtained by RNA-seq.(JPG 125 kb) [file 12864_2019_6036_MOESM3_ESM.jpg]

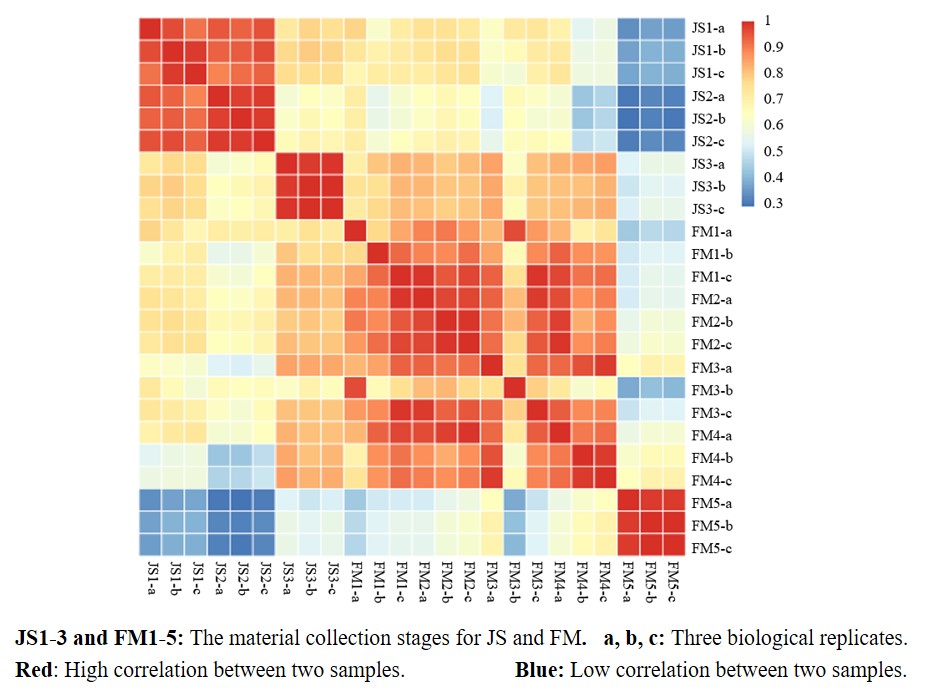

Supplement: Supplementary file 4 — The correlation heatmap of all samples in JS and FM cultivar.(JPG 123 kb) [file 12864_2019_6036_MOESM4_ESM.jpg]

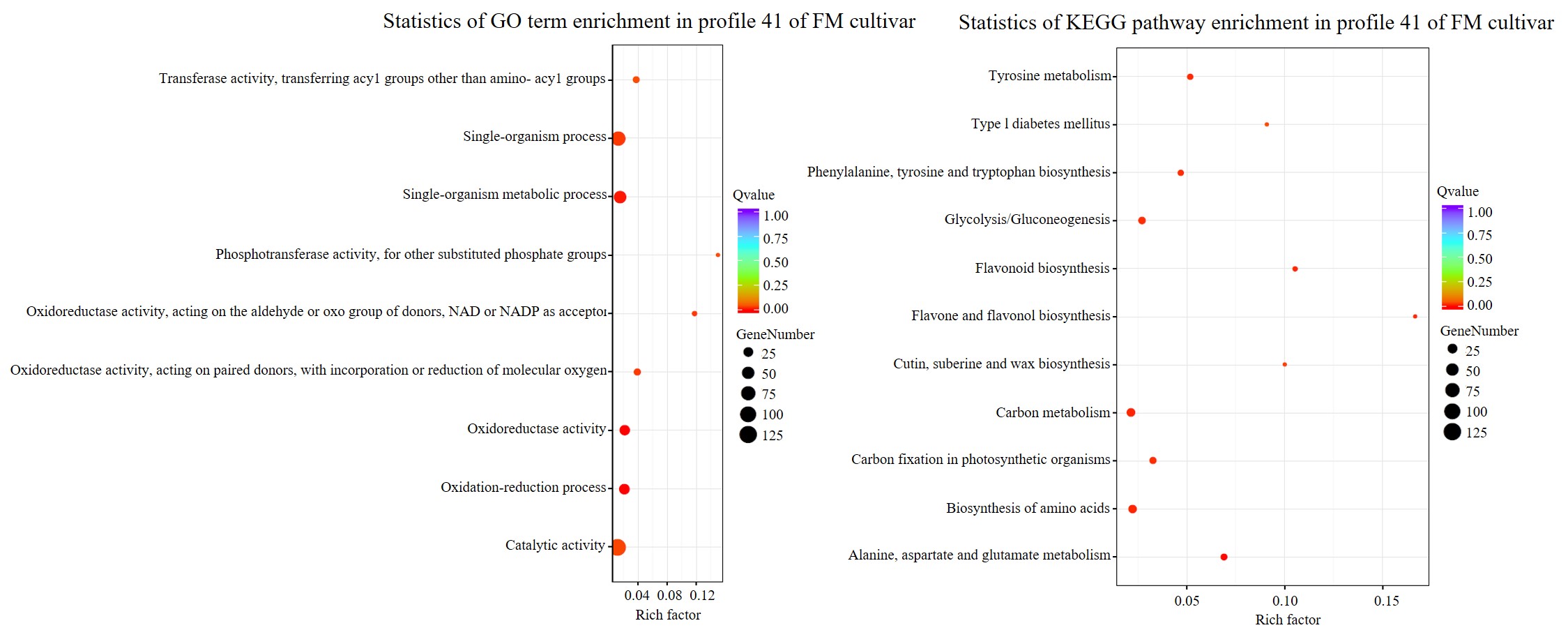

Supplement: Supplementary file 5 — GO term and KEGG pathway enrichment statistics of DEGs in profile 41 of FM cultivar.(JPG 239 kb) [file 12864_2019_6036_MOESM5_ESM.jpg]

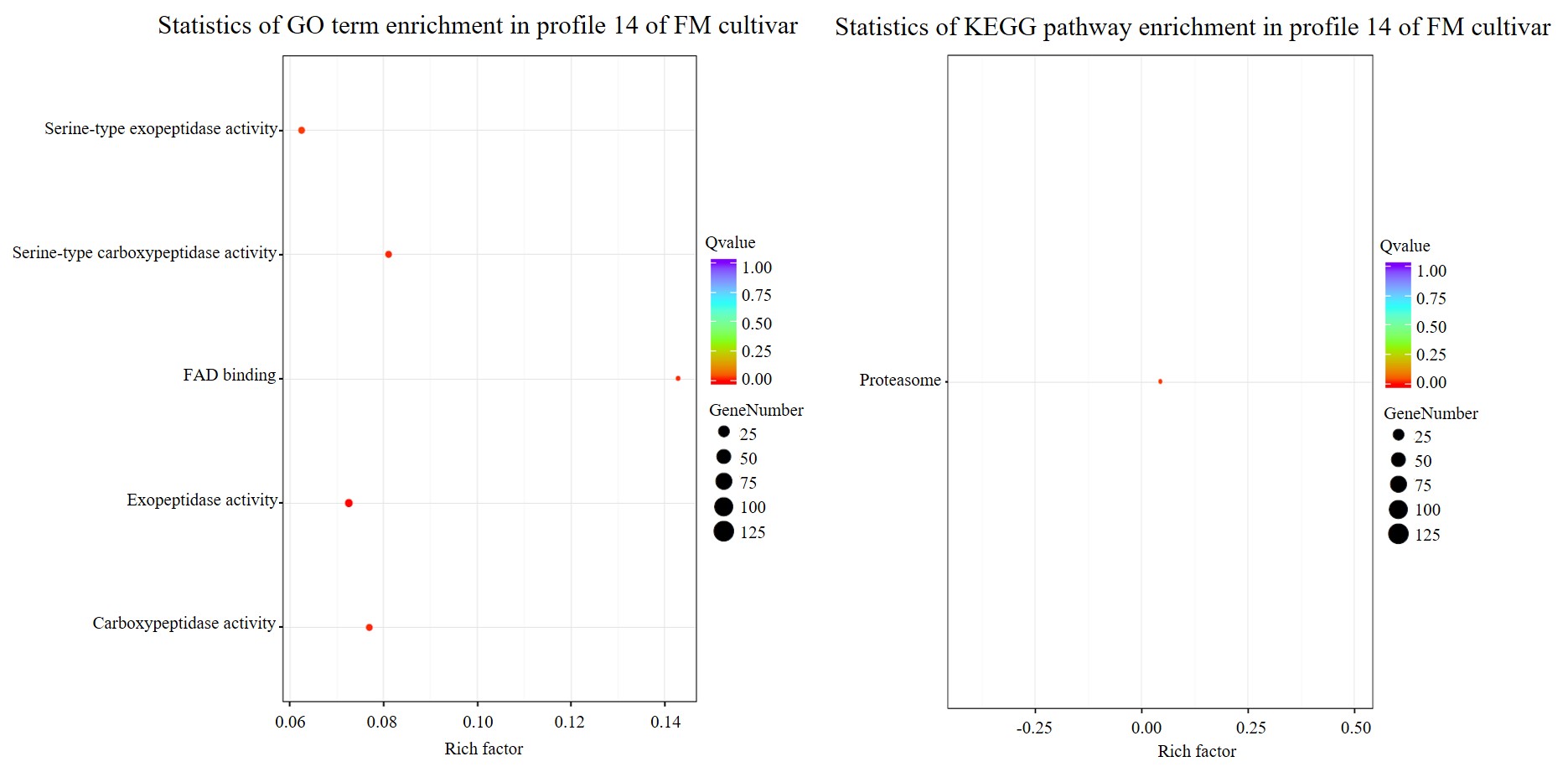

Supplement: Supplementary file 7 — GO term and KEGG pathway enrichment statistics of DEGs in profile 14 of FM cultivar.(JPG 138 kb) [file 12864_2019_6036_MOESM7_ESM.jpg]

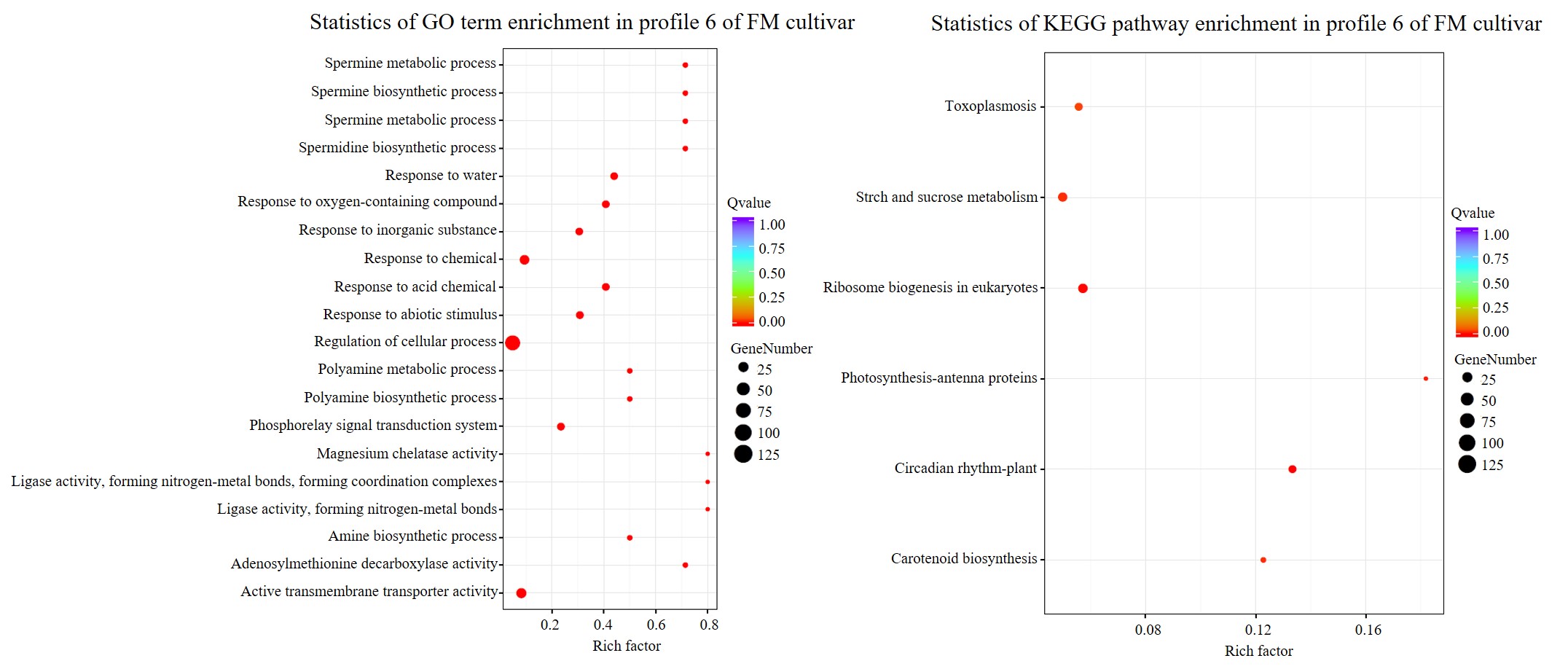

Supplement: Supplementary file 8 — GO term and KEGG pathway enrichment statistics of DEGs in profile 6 of FM cultivar.(JPG 248 kb) [file 12864_2019_6036_MOESM8_ESM.jpg]

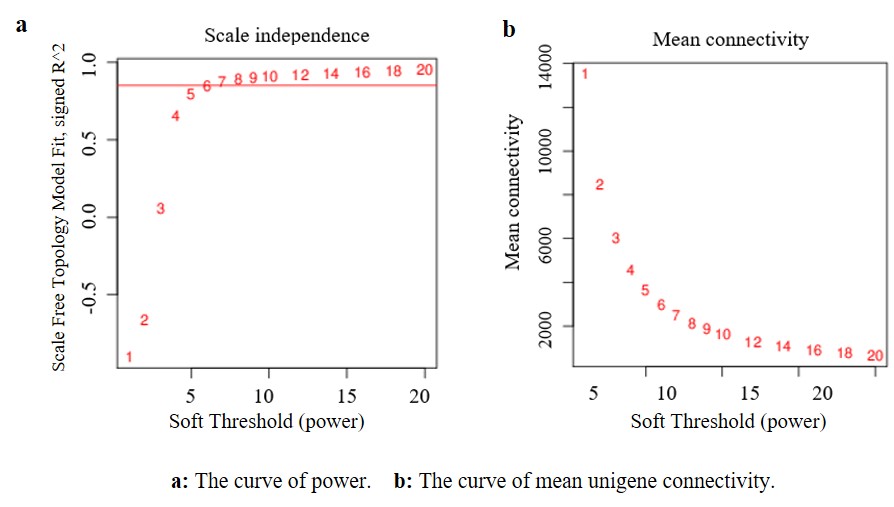

Supplement: Supplementary file 12 — Filtering of power value for gene network weight analysis.(JPG 54 kb) [file 12864_2019_6036_MOESM12_ESM.jpg]

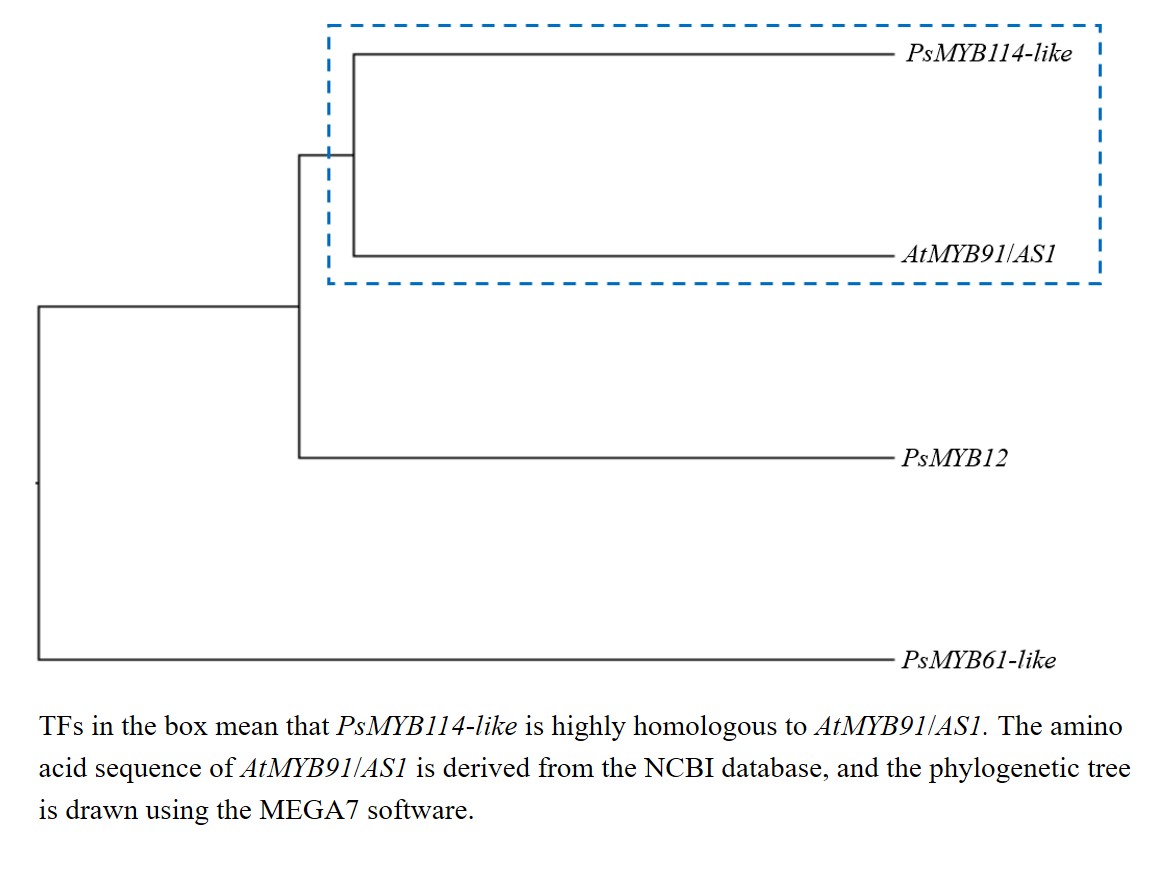

Supplement: Supplementary file 15 — Phylogenetic analysis of MYB-like DEGs in P. rockii and AtMYB91/AS1 in A. thaliana.(JPG 95 kb) [file 12864_2019_6036_MOESM15_ESM.jpg]
